# COVID-19 severity and early inflammatory biomarkers in patients with immune-mediated inflammatory rheumatic diseases: a multicenter cohort study

**DOI:** 10.25122/jml-2026-0098

**Published:** 2026-08

**Authors:** Andreea-Iulia Vlădulescu-Trandafir, Gelu Onose, Violeta-Claudia Bojincă, Andreea-Valentina Suciu, Sebastian Giuvară, Constantin Munteanu, Corneliu-Dan Blendea, Cristina Popescu

**Affiliations:** 1 Faculty of Medicine, Carol Davila University of Medicine and Pharmacy, Bucharest, Romania; 2 Neuromuscular Rehabilitation Clinic Division, Bagdasar-Arseni Teaching Emergency Hospital, Bucharest, Romania; 3 Department of Internal Medicine and Rheumatology, Sfânta Maria Hospital, Bucharest, Romania; 4 Faculty of Medical Bioengineering, Grigore T. Popa University of Medicine and Pharmacy, Iași, Romania; 5 Clinical Regional Emergency Hospital Ilfov, Bucharest, Romania; 6 Faculty of Medicine, Titu Maiorescu University, Bucharest, Romania

**Keywords:** COVID-19, SARS-CoV-2, inflammatory biomarkers, immune-mediated inflammatory rheumatic diseases, cohort studies, rheumatic diseases, AISI, aggregate index of systemic inflammation, ALT, alanine aminotransferase, AST, aspartate aminotransferase, axSpA, axial spondyloarthritis, bDMARD, biologic disease-modifying antirheumatic drug, CI, confidence interval, CK, creatine kinase, COVID-19, coronavirus disease 2019, CRP, C-reactive protein, csDMARD, conventional synthetic disease-modifying antirheumatic drug, DMARD, disease-modifying antirheumatic drug, dNLR, derived neutrophil-to-lymphocyte ratio, ESR, erythrocyte sedimentation rate, GCP, Good Clinical Practice, GDPR, General Data Protection Regulation, GOT, glutamic-oxaloacetic transaminase, HCT, hematocrit, HR, hazard ratio, IL-6, interleukin-6, ILD, interstitial lung disease, IMIRD, immune-mediated inflammatory rheumatic disease, IQR, interquartile range, IRR, incidence rate ratio, JAK, Janus kinase, NET, neutrophil extracellular trap, NLR, neutrophil-to-lymphocyte ratio, OR, odds ratio, RA, rheumatoid arthritis, RR, risk ratio, SARS-CoV-2, severe acute respiratory syndrome coronavirus 2, SD, standard deviation, SIRI, systemic inflammation response index, SLE, systemic lupus erythematosus, SM, supplementary material, SpO_2_, peripheral oxygen saturation, SSc, systemic sclerosis, STROBE, Strengthening the Reporting of Observational Studies in Epidemiology, tsDMARD, targeted synthetic disease-modifying antirheumatic drug, WHO, World Health Organization

## Abstract

Patients with immune-mediated inflammatory rheumatic diseases (IMIRDs) may be more vulnerable to severe acute respiratory syndrome coronavirus 2 (SARS-CoV-2) infection because of underlying immune dysregulation. However, it remains unclear which factors determine coronavirus disease 2019 (COVID-19) severity in this population, especially in Eastern European settings. We conducted a retrospective, multicenter observational study including 211 patients with IMIRDs diagnosed with COVID-19 between 01.03.2020 and 31.12.2024. We collected clinical, demographic, and laboratory data within the first 72 hours of symptom onset and classified COVID-19 severity into three ordered categories. Associations with severity were examined using separate univariable ordinal logistic regression models and an additional multivariable model including age and IMIRD subtype. The main factors linked to more severe COVID-19 were older age (adjusted odds ratio [OR] 1.06; 95% confidence interval [CI]:1.03–1.09) and comorbidity burden (OR = 1.22; 95% CI, 1.12–1.31). Among patients with available laboratory measurements, higher levels of acute-phase reactants, as well as a higher derived neutrophil-to-lymphocyte ratio (dNLR) (OR = 1.30; 95% CI, 1.10–1.54) and lower monocyte counts (OR = 0.08; 95% CI, 0.02–0.36), remained associated with increased severity after Bonferroni correction for multiple comparisons. Accounting for age, patients with SLE had lower odds of greater COVID-19 severity than those with RA (OR = 0.35; 95% CI, 0.13–0.90). COVID-19 severity was associated with age, comorbidity burden, and early inflammatory response, with an age-adjusted difference also observed between SLE and RA. Routine inflammatory parameters and composite indices may contribute to early risk stratification in this population.

## Introduction

COVID-19 can be considered one of the greatest global health concerns experienced in the last century, especially for patients with chronic conditions. A particular such category is represented by individuals with immune-mediated inflammatory rheumatic diseases (IMIRDs), who are considered a potentially vulnerable population. Their susceptibility to infections and adverse outcomes can be explained by a complex interplay between disease-related immune perturbations, comorbidities, and specific treatments [1–3].

Whether patients with IMIRDs are at increased risk of severe outcomes following SARS-CoV-2 infection remains uncertain. A Danish nationwide cohort of 66,840 patients with inflammatory rheumatic diseases reported approximately twofold higher risks of severe COVID-19 (incidence rate ratio [IRR] = 2.18; 95% confidence interval [CI], 1.94–2.45) and COVID-19-related death (IRR = 1.98; 95% CI, 1.69–2.33) compared with matched population controls [4]. In the OpenSAFELY cohort, the increased risk of COVID-19-related death declined from an adjusted hazard ratio (HR) of 1.23 (95% CI, 1.20–1.27) to 1.15 (95% CI, 1.11–1.18) after additional adjustment for body mass index, cardiovascular disease, diabetes, and glucocorticoid use [5]. Conversely, the Euro-COVIMID study found that overall SARS-CoV-2 infection severity in patients with immune-mediated inflammatory diseases resembled that of the general population [6]. A meta-analysis also reported increased mortality (odds ratio [OR] 1.74; 95% CI, 1.08–2.80), but no significant differences in hospitalization, intensive care admission, or mechanical ventilation [2]. Similarly, in the SORCOM registry, the higher mortality observed among patients with systemic autoimmune conditions in univariable analyses was not accompanied by differences in overall disease severity after multivariable adjustment [7]. Moreover, in a US multicenter cohort of 9,730 patients with rheumatoid arthritis (RA), mortality risk was more than twice as high before matching (risk ratio [RR] 2.11; 95% CI, 1.90–2.34), whereas mortality, hospitalization, intensive care admission, mechanical ventilation, and severe COVID-19 no longer differed significantly from patients without RA after propensity-score matching for demographic characteristics and comorbidities [8]. This discrepancy reflects the challenge of disentangling the underlying disease effect from other COVID-19-related factors, including the inflammatory response. Data from Eastern Europe remain limited and address different aspects of COVID-19 in patients with rheumatic diseases. Recent Polish studies have discussed clinical recommendations for prevention and treatment [9] and examined the course of infection in patients with systemic autoimmune rheumatic diseases [10]. In Romania, previous research has focused on the impact of the pandemic, access to medical care, and vaccination willingness among patients with autoimmune or immune-mediated diseases [11]. In our previous work, we investigated factors associated with COVID-19 severity in patients with axial spondyloarthritis (axSpA) using data from the Romanian Registry of Rheumatic Diseases [12]. That specific analysis was limited to a single disease and used a binary severity outcome. The present study extends this line of investigation to a separate hospital-based cohort including four IMIRD subtypes and examines early clinical and laboratory findings across three ordered categories of COVID-19 severity.

Several risk factors for unfavorable COVID-19 outcomes in patients with IMIRDs have been identified, some shared with the general population – such as older age, male sex, and comorbidities including obesity, diabetes mellitus, cardiovascular diseases, and malignancies – and others IMIRD-specific, including high disease activity, interstitial lung disease (ILD), elevated baseline C-reactive protein (CRP), thrombocytosis, and severe lymphopenia [7,13,14].

There has been evidence to suggest that severe cases of COVID-19 are accompanied by a dysregulated immune response, involving neutrophil predominance, lymphocyte depletion, and elevated acute-phase reactants. Such findings may indicate an imbalance between innate immune activation and adaptive immune suppression, which has been linked to adverse clinical outcomes. Importantly, this imbalance may be captured by simple hematological parameters, such as neutrophils and lymphocytes, which represent key components of the innate and adaptive immune responses, respectively [15–17].

In this context, readily available biomarkers derived from routine complete blood counts have gained increasing attention as practical indicators of systemic inflammation and immune activation. Some composite indices, such as the neutrophil-to-lymphocyte ratio (NLR), together with other related ones, including the derived NLR (dNLR), Systemic Inflammation Response Index (SIRI), and Aggregate Index of Systemic Inflammation (AISI) – see below – provide an integrated perspective on immune status by combining these cellular components. These indices are easily accessible, cost-effective, and reproducible in clinical practice and have been associated with disease severity and mortality in the general population with COVID-19 [18–21]. However, their potential role in patients with IMIRDs, who may already exhibit baseline immune perturbations, remains incompletely explored, particularly in the context of early disease assessment.

In patients with IMIRDs, factors associated with COVID-19 severity have often been evaluated separately or included mainly as adjustment variables, rather than considered together. At the same time, many observational studies have used binary outcomes such as hospitalization or mortality, without capturing the full spectrum of infection severity. This may reduce sensitivity to intermediate stages and make it more difficult to identify early risk patterns, especially in a population with heterogeneous baseline characteristics [2,4,6,13,22–26]. An ordinal approach to disease severity may better reflect real-life decision-making and help identify individuals at risk of deterioration before severe complications occur [25,26].

Building on our previous work, which comparatively described the clinical and laboratory profiles of IMIRD versus non-patients with IMIRD with COVID-19 [27], the present study focuses on the analysis of disease severity and its determinants solely in the IMIRD cohort. By modeling severity as an ordered outcome and focusing on parameters available within the first 72 hours of symptom onset, we aimed to better understand how host-related factors, inflammatory response, comorbidity burden, and disease subtype contribute to the clinical course of SARS-CoV-2 infection in this population.

## Material and methods

### Ethical considerations

This study followed the Declaration of Helsinki, Good Clinical Practice international standards, and applicable local regulations, and was approved by the Scientific Research Ethics Committees of the participating institutions: “Sfânta Maria” Clinical Hospital, Bagdasar–Arseni Emergency Clinical Hospital, and Colentina Clinical Hospital. Patient data were anonymized using coded identifiers. All participants provided informed consent, including consent for data processing in compliance with the General Data Protection Regulation (GDPR) and for the use of electronically recorded data exclusively for medical research purposes.

### Study design and objectives

This retrospective, observational, multicentric study included 211 patients with IMIRDs, i.e., RA, systemic lupus erythematosus (SLE), systemic sclerosis (SSc), and axSpA, all of whom tested positive for SARS-CoV-2 infection during 1 March 2020–31 December 2024. Patients were recruited from the three aforementioned clinical hospitals in Bucharest.

The main objective of this study was to identify clinical and paraclinical factors associated with progression to severe COVID-19 among patients with IMIRDs, with particular attention to early inflammatory markers.

For full transparency, we report that the IMIRD cohort (*n* = 211) was previously described in a separate analysis comparing demographic, clinical, and laboratory characteristics between IMIRD and non-IMIRD patients [27]. This study extends this dataset by investigating clinical and early laboratory factors associated with three ordered categories of COVID-19 severity in the IMIRD cohort only, using an ordinal modeling approach.

### Eligibility criteria

Inclusion criteria comprised adults (age ≥ 18 years) with a diagnosis of RA, SLE, SSc, or axSpA who had a confirmed SARS-CoV-2 infection (by positive reverse transcription-polymerase chain reaction or rapid antigen test) between March 2020 and December 2024. We collected clinical and paraclinical data within the first 72 hours after symptom onset to ensure uniformity and keep the dataset comparable, given that biochemical parameters and clinical features change over the course of infection. If no measurement was available within this interval, we recorded the corresponding value as missing.

Exclusion criteria: (i) another infection at presentation (other than SARS-CoV-2) that might have influenced paraclinical parameters; (ii) incomplete or inconsistent information concerning the timing of symptom onset and/or outcomes; (iii) when the available medical records did not permit reliable determination of infection timing, assignment of the COVID-19 severity category, or ascertainment of the clinical outcome; and (iv) the inability or unwillingness to give informed consent.

Available information on background antirheumatic and immunomodulatory treatment was retrieved from the medical records. For descriptive purposes, therapies were grouped as conventional synthetic disease-modifying antirheumatic drugs (DMARDs) and immunosuppressive agents, targeted synthetic DMARDs (Janus kinase [JAK] inhibitors), and biologic DMARDs according to therapeutic target or mechanism. Background glucocorticoid use was recorded separately from corticosteroids prescribed specifically for COVID-19. Treatment frequencies according to IMIRD subtype are presented in Supplementary Material 1. These variables were not included in the regression models because dose, treatment duration, adherence, and continuation or interruption after COVID-19 onset could not be reconstructed consistently. Moreover, corticosteroids prescribed for acute COVID-19 were administered in response to the evolving clinical course and could not be evaluated as baseline predictors without substantial confounding by indication and reverse causality. A comprehensive analysis of DMARDs by molecule type, use period, and treatment lines (first/second DMARDs, etc.) would require greater characterization, beyond the scope of the present study.

Supplementary Material 1

All eligible patients during the study period were included. Because this was a retrospective study, we did not perform an a priori sample size calculation.

### Data sources and variables

Data collection followed a standardized protocol implemented across all participating hospitals, using identical case report forms and interview instruments, applied by the same investigator. Data were aggregated from patients’ medical records, institutional hospital databases, and, when required, directly from patients through face-to-face or telephone interviews, following the obtaining of their respective written informed consent.

A broad set of variables was recorded and analyzed descriptively, including demographic characteristics, clinical parameters, laboratory findings, and comorbid conditions. Among paraclinical variables, we included peripheral oxygen saturation (SpO_2_), as well as laboratory tests: features of systemic inflammation [CRP, erythrocyte sedimentation rate (ESR), fibrinogen, ferritin, and interleukin-6 (IL-6)], coagulation parameters (D-dimers and coagulation times), complete blood count, inflammation indices described below (NLR, dNLR, SIRI, AISI), procalcitonin, renal function (creatinine, urea), hepatic function [alanine aminotransferase (ALT), aspartate aminotransferase (AST)], myocardial and muscle injury markers [creatine kinase (CK) and CK-MB], and vitamin D levels. However, as laboratories in different hospitals did not have the same test availability, certain biomarkers – ferritin, IL-6, CK-MB and vitamin D – were available for only 13 patients in total and were therefore excluded from statistical modeling to preserve data homogeneity.

The following composite inflammatory indices were calculated based on routine hematological parameters:

- NLR, defined as the ratio between absolute neutrophil and lymphocyte counts;
- dNLR, calculated as the neutrophil count divided by the difference between total leukocyte count and neutrophil count;
- SIRI, determined as (neutrophil count × monocyte count) / lymphocyte count;
- AISI, calculated as (neutrophil count × monocyte count × platelet count) / lymphocyte count.

However, despite the fact that laboratories performed their analyses locally, all centers relied on standardized platforms with comparable analytical techniques and reference intervals, allowing for acceptable consistency between centers.

### Statistical analysis

All analyses were performed with R, version 4.4.1 (R Foundation for Statistical Computing, Vienna, Austria) [28]. Continuous variables are reported as mean ± standard deviation (SD), whereas categorical variables are presented as absolute counts and relative frequencies (%). Statistical significance was defined as a two-sided *P* < 0.05, corresponding to 95% confidence intervals (CIs).

To address the primary objective of the study – identification of clinical and paraclinical factors associated with COVID-19 severity – an ordinal logistic regression model was applied. The primary outcome was COVID-19 severity, classified according to World Health Organization (WHO) criteria into three ordered categories: mild (C1), moderate (C2), and severe/critical (C3). With a relatively low number of critical patients, the classification combined severe and critical infections into a single category (C3) to ensure model stability and avoid sparse-data bias.

COVID-19 severity was treated as an ordinal dependent variable, reflecting a hierarchy of clinical states (C2 more severe than C1; C3 more severe than C2), without assuming equal distances between categories. The regression model included clinical, paraclinical, and demographic characteristics of the patients in the study. The ordinal logistic regression model was specified using cumulative logits, as follows: for a dependent variable Y representing COVID-19 severity (Y ∈ {1, 2, 3}) and a predictor X (e.g., lymphocytes), the model estimates:

logit[P(Y ≤ 1 | X)] = α_1_ + βX and

logit[P(Y ≤ 2 | X)] = α_2_ + βX,

where α_1_ and α_2_ are the threshold (intercept) parameters for the severity cut points (denoted by “1|2” and “2|3” in the results tables), and β is the regression coefficient for the predictor of interest. The proportional odds assumption implies that the effect of X is constant across severity thresholds.

The exponentiation of β will give the odds ratio (OR), i.e., the change in the odds of the transition to the next level of severity category per unit increase in predictor X. Model performance was additionally evaluated via the value of Nagelkerke’s *R*^2^, which reflects the proportion of variance in the dependent variable explained by the model, with values ranging from 0 (no explanatory power) to 1 (maximum explanatory power).

Model-predicted probability curves were used to illustrate the continuous associations between each predictor and the three severity categories. Curve intersections indicate values at which the model-predicted probabilities of two severity categories become equal. For laboratory biomarkers, approximate intersection values were reported only when the corresponding association remained statistically significant after Bonferroni correction. Curve intersections and regions of more rapid probability change were not evaluated using a dedicated cut-point analysis and were therefore not considered validated clinical thresholds.

Each biomarker-specific analysis included all patients with an available measurement for that variable; consequently, the analytical sample size differed between models. Missing values were not imputed. The extent of missing laboratory data was summarized in the overall cohort and separately within each COVID-19 severity category.

Given the exploratory aim of the study, associations between COVID-19 severity and clinical, demographic, and laboratory variables were primarily examined using separate univariable ordinal logistic regression models, each including COVID-19 severity as the outcome and one predictor at a time. To assess the association between IMIRD subtype and COVID-19 severity while accounting for age, we fitted an additional multivariable ordinal logistic regression model including age and IMIRD subtype, with RA as the reference category.

More extensive ordinal regression models incorporating additional clinical covariates and laboratory variables were explored. However, the simultaneous inclusion of age, sex, SpO_2_, comorbidity burden, IMIRD subtype, and the biomarkers that remained significant after correction for multiple comparisons did not yield estimable coefficient values. The substantial reduction in the complete-case sample, the limited number of patients in the higher severity categories, and the overlap among inflammatory biomarkers precluded reliable estimation of the fully adjusted model. Therefore, only models that produced stable and interpretable estimates are reported.

To account for multiple comparisons across the 20 laboratory parameters evaluated in the univariable ordinal regression analyses, a Bonferroni correction was performed as a sensitivity analysis. Adjusted *P* values were calculated by multiplying each raw *P* value by 20, with values exceeding 1.00 capped at 1.00. A Bonferroni-adjusted *P* value < 0.05 was considered statistically significant. The study was conducted in accordance with the Strengthening the Reporting of Observational Studies in Epidemiology (STROBE) Statement (see Supplementary Material 2).

Supplementary Material 2

## Results

### Demographic characteristics and COVID-19 severity

The study population included 211 patients with IMIRDs, whose baseline demographic and clinical characteristics have been previously described in detail [27]. Briefly, the cohort comprised patients with RA (*n* = 90), axSpA (*n* = 46), SLE (*n* = 45), and SSc (*n* = 30) and was predominantly female (76%), with a mean age of 54 ± 14 years. Most patients (90%) were non-smokers. In this analysis, we further examined these characteristics in relation to COVID-19 severity. To improve readability, additional tables and figures of complementary analyses and secondary results are provided in Supplementary Material 3 (SM3).

Supplementary Material 3

Despite the common association of male sex with more severe COVID-19, there were no statistically significant associations between sex and SARS-CoV-2 infection severity among patients with IMIRD, although the predominantly female composition of the cohort (76%) may have limited the ability to detect sex-related differences. Ordinal logistic regression showed that being male did not increase the odds of progressing to a more severe illness state (OR = 1.03; 95% CI, 0.54–1.96; *P* = 0.930), as shown in SM3 Table S1 and Figure S1.

In contrast, age was significantly associated with COVID-19 severity (OR = 1.06; 95% CI, 1.04–1.09; *P* < 0.001) (Table 1). Increasing age was significantly associated with higher odds of more severe SARS-CoV-2 infection, confirming age as an important non-modifiable risk factor in this cohort. The graphs of predicted probabilities (Figure 1) demonstrate that the majority of patients under 60 years old had mild symptoms (C1). On the contrary, patients aged 60 or older had a high risk of severe infection.

**Table 1:** Ordinal logistic regression analysis of the association between age and COVID-19 severity

Parameter	OR	95% CI	*P*
1|2	53.01	13.04–215.41	**< 0.001**
2|3	146.48	33.95–631.91	**< 0.001**
Age (years)	1.06	1.04–1.09	**< 0.001**
Observations (*n*)	211		
Nagelkerke *R*^2^	0.155		

Estimates are odds ratios from a univariable ordinal logistic regression model with COVID-19 severity as the ordered outcome (C1, mild; C2, moderate; C3, severe/critical). Rows 1|2 and 2|3 are the threshold (intercept) parameters for the severity cut-points. Bold P values indicate statistical significance (*P* < 0.05). CI, confidence interval; *n*, number of patients contributing to the model; OR, odds ratio.

**Figure 1:**
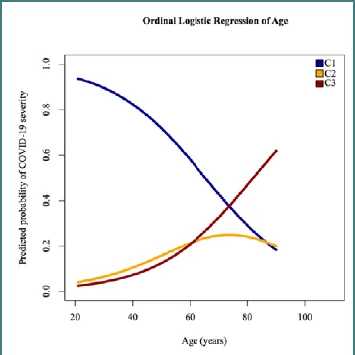
Model-predicted probabilities of COVID-19 severity categories across age. Curves are derived from the univariable ordinal logistic regression model in Table 1. C1, mild; C2, moderate; C3, severe/critical.

No statistically significant association was observed between COVID-19 severity and the patients’ environment of origin (OR = 1.78; 95% CI, 0.90–3.50; *P* = 0.097) and smoking status (OR = 2.09; 95% CI, 0.75–5.84; *P* = 0.160). For further reference, please see SM3 Tables S2 and S3 and Figures S2 and S3.

### Paraclinical findings and COVID-19 severity

SpO_2_ on admission served as an indirect parameter for lung involvement. Low initial SpO_2_ values were associated with an increased likelihood of more severe infection (OR = 0.45; 95% CI, 0.37–0.54; *P* < 0.001). Additional information can be found in SM3 Table S4.

Figure 2 shows the corresponding model-predicted probabilities across SpO_2_ values. The curves changed steeply within the low-90% range; however, this pattern was not assessed as a diagnostic threshold and should not be interpreted as a validated clinical cut-off.

**Figure 2:**
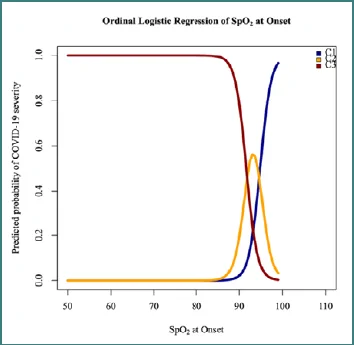
Model-predicted probabilities of COVID-19 severity categories across SpO_2_ values. C1, mild; C2, moderate; C3, severe/critical; SpO_2_, peripheral oxygen saturation.

Laboratory data availability varied across the evaluated parameters and severity categories. Missing observations were generally more frequent in C1. Detailed counts and proportions of missing values for all laboratory variables are presented in Supplementary Material 4.

Supplementary Material 4

### Inflammatory markers

Statistical analysis revealed a positive association between COVID-19 severity and markers of systemic inflammation. ESR levels showed a significant association with severity (OR = 1.03; 95% CI, 1.01–1.04; *P* = 0.001), as seen in Table 2. The predicted probability of severe or critical disease increased progressively with higher ESR values, with an approximate intersection of the probability curves around 60 mm/h (Figure 3).

**Table 2:** Ordinal logistic regression analysis of the association between ESR and COVID-19 severity

Parameter	OR	95% CI	*P*
1|2	2.63	1.25–5.54	**0.012**
2|3	9.67	3.98–23.50	**< 0.001**
ESR (mm/h)	1.03	1.01–1.04	**0.001**
Observations (*n*)	81		
Nagelkerke *R*^2^	0.181		

Estimates are odds ratios from a univariable ordinal logistic regression model with COVID-19 severity as the ordered outcome (C1, mild; C2, moderate; C3, severe/critical). Rows 1|2 and 2|3 are the threshold (intercept) parameters for the severity cut-points. Bold P values indicate statistical significance (*P* < 0.05). CI, confidence interval; *n*, number of patients contributing to the model; OR, odds ratio; ESR, erythrocyte sedimentation rate.

**Figure 3:**
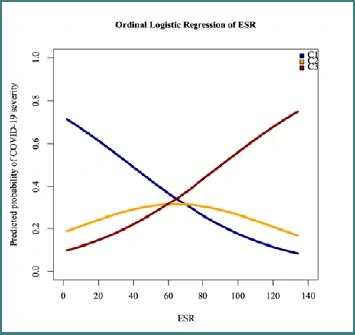
Model-predicted probabilities of COVID-19 severity categories across ESR values. C1, mild; C2, moderate; C3, severe/critical; ESR, erythrocyte sedimentation rate.

Table 3 shows that for CRP, each unit increase was associated with higher odds of severe disease (OR = 1.02; 95% CI, 1.01–1.03; *P* < 0.001). The predicted probability of severe or critical disease increased progressively with higher CRP values, with an approximate curve intersection around 100 mg/L (Figure 4).

**Table 3:** Ordinal logistic regression analysis of the association between CRP and COVID-19 severity

Parameter	OR	95% CI	*P*
1|2	2.62	1.53–4.50	**0.001**
2|3	9.14	4.63–18.05	**< 0.001**
CRP (mg/L)	1.02	1.01–1.03	**< 0.001**
Observations (*n*)	108		
Nagelkerke *R*^2^	0.266		

Estimates are odds ratios from a univariable ordinal logistic regression model with COVID-19 severity as the ordered outcome (C1, mild; C2, moderate; C3, severe/critical). Rows 1|2 and 2|3 are the threshold (intercept) parameters for the severity cut-points. Bold *P* values indicate statistical significance (*P* < 0.05). CI, confidence interval; *n*, number of patients contributing to the model; OR, odds ratio; CRP, C-reactive protein.

**Figure 4:**
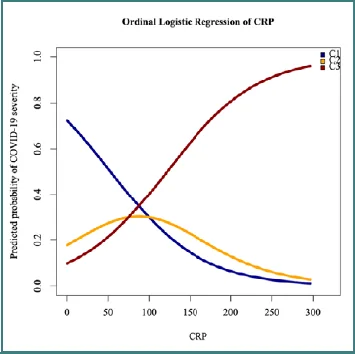
Model-predicted probabilities of COVID-19 severity categories across CRP values. C1, mild; C2, moderate; C3, severe/critical; CRP, C-reactive protein.

Fibrinogen levels were also associated with increased severity, although the effect size was smaller (OR = 1.01; 95% CI, 1.00–1.01; *P* = 0.002). This data is detailed in Table 4. Similarly, as for ESR and CRP, the model-predicted probability of C3 disease increased progressively with higher fibrinogen values; the descriptive intersection of the probability curves was around 400 mg/dL for this cohort.

**Table 4:** Ordinal logistic regression analysis of the association between fibrinogen and COVID-19 severity

Parameter	OR	95% CI	*P*
1|2	6.85	1.20–39.09	**0.031**
2|3	26.22	4.04–170.13	**0.001**
Fibrinogen (mg/dL)	1.01	1.00–1.01	**0.002**
Observations (*n*)	59		
Nagelkerke *R*^2^	0.216		

Estimates are odds ratios from a univariable ordinal logistic regression model with COVID-19 severity as the ordered outcome (C1, mild; C2, moderate; C3, severe/critical). Rows 1|2 and 2|3 are the threshold (intercept) parameters for the severity cut-points. Bold *P* values indicate statistical significance (*P* < 0.05). CI, confidence interval; *n*, number of patients contributing to the model; OR, odds ratio.

### Leukocyte subpopulations

Lower baseline lymphocyte levels were associated with increased disease severity (OR = 0.40; 95% CI, 0.22–0.72; *P* = 0.003; Table 5). Model-predicted probability curves showed that lower lymphocyte values were associated with a higher probability of C3, whereas higher lymphocyte levels were associated with a shift toward C1 and C2 (Figure 6).

**Table 5:** Ordinal logistic regression analysis of the association between lymphocyte count and COVID-19 severity

Parameter	OR	95% CI	*P*
1|2	0.33	0.15–0.74	**0.008**
2|3	0.89	0.42–1.93	0.774
Lymphocytes (×10^9^/L)	0.40	0.22–0.72	**0.003**
Observations (*n*)	110		
Nagelkerke *R*^2^	0.113		

Estimates are odds ratios from a univariable ordinal logistic regression model with COVID-19 severity as the ordered outcome (C1, mild; C2, moderate; C3, severe/critical). Rows 1|2 and 2|3 are the threshold (intercept) parameters for the severity cut-points. Bold *P* values indicate statistical significance (*P* < 0.05). CI, confidence interval; *n*, number of patients contributing to the model; OR, odds ratio.

**Figure 5:**
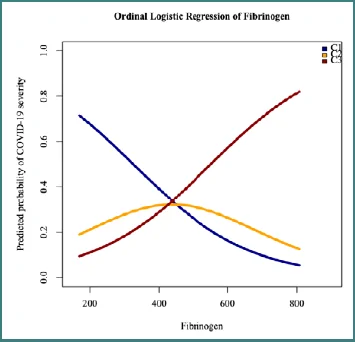
Model-predicted probabilities of COVID-19 severity categories across fibrinogen values. C1, mild; C2, moderate; C3, severe/critical.

**Figure 6:**
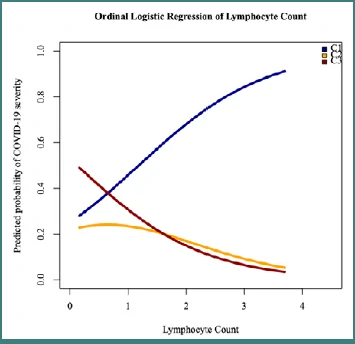
Model-predicted probabilities of COVID-19 severity categories across lymphocyte values. C1, mild; C2, moderate; C3, severe/critical.

The monocyte count was also significantly associated with COVID-19 severity, with lower values observed in patients with more severe disease (OR = 0.08; 95% CI, 0.02–0.36; *P* = 0.001; Table 6). The probability curves suggested a shift toward higher probabilities of severe or critical disease (C3) at lower monocyte levels, while higher values were more frequently associated with milder clinical presentations (Figure 7).

**Table 6:** Ordinal logistic regression analysis of the association between monocyte count and COVID-19 severity

Parameter	OR	95% CI	*P*
1|2	0.24	0.10–0.60	**0.003**
2|3	0.64	0.26–1.53	0.309
Monocytes (×10^9^/L)	0.08	0.02–0.36	**0.001**
Observations (*n*)	108		
Nagelkerke *R*^2^	0.140		

Estimates are odds ratios from a univariable ordinal logistic regression model with COVID-19 severity as the ordered outcome (C1, mild; C2, moderate; C3, severe/critical). Rows 1|2 and 2|3 are the threshold (intercept) parameters for the severity cut-points. Bold *P* values indicate statistical significance (*P* < 0.05). CI, confidence interval; *n*, number of patients contributing to the model; OR, odds ratio.

**Figure 7:**
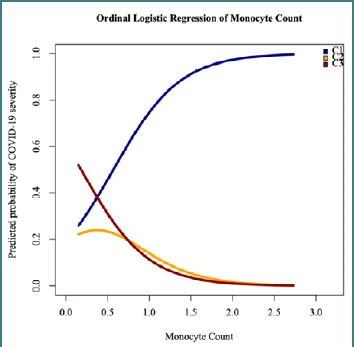
Model-predicted probabilities of COVID-19 severity categories across monocyte values. C1, mild; C2, moderate; C3, severe/critical.

Absolute eosinophil count was also significantly associated with COVID-19 severity. Lower eosinophil levels were observed in patients with more severe disease (OR = 0.50; 95% CI, 0.30–0.86; *P* = 0.012; SM3 Table S5).

### Composite inflammatory indices

Composite inflammatory indices capturing the balance between innate and adaptive immune responses showed differential associations with COVID-19 severity. Both the NLR and dNLR were associated with increased odds of more severe infection in the univariable analyses (NLR: OR = 1.10; 95% CI, 1.03–1.18; *P* = 0.006; dNLR: OR = 1.30; 95% CI, 1.10–1.54; *P* = 0.002), as depicted in Tables 7 and 8.

**Table 7:** Ordinal logistic regression analysis of the association between NLR and COVID-19 severity

Parameter	OR	95% CI	*P*
1|2	2.19	1.26–3.81	**0.006**
2|3	5.45	2.89–10.24	**< 0.001**
NLR	1.10	1.03–1.18	**0.006**
Observations (*n*)	108		
Nagelkerke *R*^2^	0.139		

Estimates are odds ratios from a univariable ordinal logistic regression model with COVID-19 severity as the ordered outcome (C1, mild; C2, moderate; C3, severe/critical). Rows 1|2 and 2|3 are the threshold (intercept) parameters for the severity cut-points. Bold *P* values indicate statistical significance (*P* < 0.05). CI, confidence interval; *n*, number of patients contributing to the model; OR, odds ratio; NLR, neutrophil-to-lymphocyte ratio.

**Table 8:** Ordinal logistic regression analysis of the association between dNLR and COVID-19 severity

Parameter	OR	95% CI	*P*
1|2	2.87	1.50–5.52	**0.002**
2|3	7.34	3.51–15.39	**< 0.001**
dNLR	1.30	1.10–1.54	**0.002**
Observations (*n*)	108		
Nagelkerke *R*^2^	0.171		

Estimates are odds ratios from a univariable ordinal logistic regression model with COVID-19 severity as the ordered outcome (C1, mild; C2, moderate; C3, severe/critical). Rows 1|2 and 2|3 are the threshold (intercept) parameters for the severity cut-points. Bold *P* values indicate statistical significance (*P* < 0.05). CI, confidence interval; *n*, number of patients contributing to the model; OR, odds ratio; dNLR, derived neutrophil-to-lymphocyte ratio.

Lower values of these indices were predominantly associated with mild infection (C1), whereas progressive increases corresponded to a marked shift toward severe clinical phenotypes. More specifically, at lower NLR values, the predicted probability of mild disease (C1) was high, whereas the probabilities of moderate (C2) and severe (C3) disease were low.

As NLR values increased – reflecting neutrophilia and/or lymphopenia – the probability of severe COVID-19 rose sharply (Figure 8). Likewise, the presence of an elevated dNLR, which reflects neutrophil predominance relative to other circulating leukocytes, was associated with a high risk of severe illness, as illustrated by the intersection of predicted probability curves (Figure 9). For dNLR, the model-predicted probability curves showed an approximate intersection around 7. This value was considered descriptive and should not be interpreted as a validated clinical threshold.

**Figure 8:**
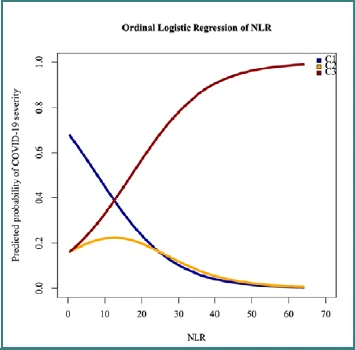
Model-predicted probabilities of COVID-19 severity categories according to NLR values. C1, mild; C2, moderate; C3, severe/critical; NLR, neutrophil-to-lymphocyte ratio.

**Figure 9:**
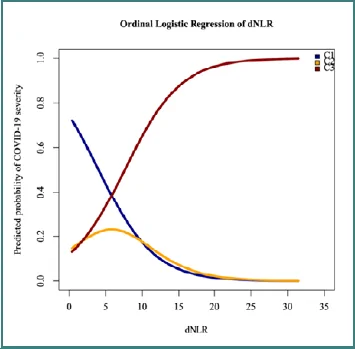
Model-predicted probabilities of COVID-19 severity categories according to dNLR values. C1, mild; C2, moderate; C3, severe/critical; dNLR, derived neutrophil-to-lymphocyte ratio.

In contrast to NLR and dNLR, no statistically significant associations with COVID-19 severity were observed for SIRI or AISI (*P* = 0.701 and *P* = 0.533, respectively; SM3 Tables S6 and S7).

### Other markers

Lower hematocrit (HCT) values were more frequently observed in patients with severe COVID-19 (OR = 0.91; 95% CI, 0.85–0.98; *P* = 0.010; SM3 Table S8). As shown in SM3 Figure S4, the probability of severe disease steadily increased as HCT declined, with progressively higher predicted probabilities at lower HCT values (SM3 Figure S4).

As one of the indicators of liver injury, the serum level of aspartate aminotransferase (AST), also known as glutamic-oxaloacetic transaminase (GOT), was significantly associated with the severity of COVID-19 (OR = 1.02; 95% CI, 1.00–1.03; *P* = 0.010; SM3 Table S9). The model-predicted probability of severe or critical disease increased with higher AST values (SM3 Figure S5).

### Paraclinical parameters without a significant association

No statistically significant association with COVID-19 severity was observed for procalcitonin (*P* = 0.180), D-dimers (*P* = 0.909), hemoglobin levels (*P* = 0.112), platelet count (*P* = 0.316), total leukocyte count (*P* = 0.746), alanine aminotransferase (ALT) (*P* = 0.411), creatine kinase (CK) (*P* = 0.755) or serum creatinine levels (*P* = 0.816), as detailed in SM3 Tables S10–S17.

Bonferroni correction

After Bonferroni correction for the laboratory parameters evaluated, the associations with ESR (adjusted *P* = 0.020), CRP (adjusted *P* < 0.020), fibrinogen (adjusted *P* = 0.040), monocyte count (adjusted *P* = 0.020), and dNLR (adjusted *P* = 0.040) remained statistically significant. The associations with lymphocyte count, eosinophil count, NLR, HCT, and AST no longer met the corrected threshold for statistical significance. Complete raw and Bonferroni-adjusted *P* values are presented in Supplementary Material 5.

Supplementary Material 5

### Vaccination status and COVID-19 severity

In an exploratory univariable ordinal logistic regression analysis, unvaccinated status was associated with higher odds of greater COVID-19 severity compared with vaccinated status (OR = 2.04; 95% CI, 1.04–4.00; *P* = 0.039; Table 9). The model had limited explanatory performance (Nagelkerke *R*^2^ = 0.026). Model-predicted probabilities are shown in Figure 10. This nuance is further discussed in the Discussion.

**Table 9:** Ordinal logistic regression analysis of the association between vaccination status and COVID-19 severity

Parameter	OR	95% CI	*P*
1|2	3.04	1.67–5.53	< 0.001
2|3	7.65	4.02–14.59	< 0.001
Unvaccinated (vs vaccinated)	2.04	1.04–4.00	0.039
Observations (*n*)	211		
Nagelkerke *R*^2^	0.026		

Estimates are odds ratios from a univariable ordinal logistic regression model with COVID-19 severity as the ordered outcome (C1, mild; C2, moderate; C3, severe/critical). Rows 1|2 and 2|3 are the threshold (in-tercept) parameters for the severity cut-points. Bold *P* values indicate statistical significance (*P* < 0.05). CI, confidence interval; *n*, number of patients contributing to the model; OR, odds ratio.

**Figure 10:**
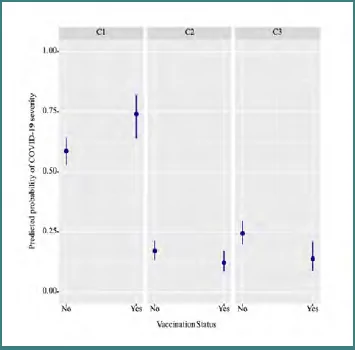
Predicted probabilities of COVID-19 severity categories stratified by vaccination status. Points are predicted probabilities and vertical bars the 95% confidence intervals. C1, mild; C2, moderate; C3, severe/critical.

### Immunomodulatory and immunosuppressive treatments

Background treatment varied across IMIRD subtypes. At least one csDMARD or immunosuppressive agent was recorded in 75.6% of patients with RA, 88.9% of those with SLE, 23.9% of those with axSpA, and 66.7% of those with SSc. Targeted synthetic DMARDs were recorded only in the RA group (14.4%), whereas bDMARD use was most frequent in axSpA (60.9%), followed by RA (32.2%), SLE (13.3%), and SSc (13.3%). Background glucocorticoids were recorded in 38.9% of patients with RA, 55.6% of those with SLE, none of those with axSpA, and 26.7% of those with SSc. Corticosteroids prescribed specifically for COVID-19 were recorded separately in 72/211 patients (34.1%). Detailed frequencies are presented in Supplementary Material 1.

### Comorbidities and COVID-19 severity

It is well established that pre-existing conditions increase the likelihood of adverse health consequences in patients with SARS-CoV-2. In our cohort, the presence of comorbidities included malignancies, as well as cardiovascular, respiratory, metabolic, musculoskeletal, neuropsychiatric, and uro-renal diseases.

The number of comorbidities was significantly associated with the C3 disease category. The greater number of comorbidities was significantly associated with the probability of contracting severe forms of the disease (OR = 1.22; 95% CI, 1.12–1.31; *P* < 0.001; Table 10). As shown in Figure 11, patients without associated pathologies had a probability of more than 80% for C1 disease. On the contrary, the probability of developing severe infection significantly increased among patients with more than 10 comorbidities.

**Table 10:** Association between the burden of comorbidities and COVID-19 severity

Parameter	OR	95% CI	*P*
1|2	5.24	3.05–9.01	**< 0.001**
2|3	14.46	7.78–26.86	**< 0.001**
Number of comorbidities	1.22	1.12–1.31	**< 0.001**
Observations (*n*)	211		
Nagelkerke *R*^2^	0.145		

Estimates are odds ratios from a univariable ordinal logistic regression model with COVID-19 severity as the ordered outcome (C1, mild; C2, moderate; C3, severe/critical). Rows 1|2 and 2|3 are the threshold (intercept) parameters for the severity cut-points. Bold *P* values indicate statistical significance (*P* < 0.05). CI, confidence interval; *n*, number of patients contributing to the model; OR, odds ratio.

**Figure 11:**
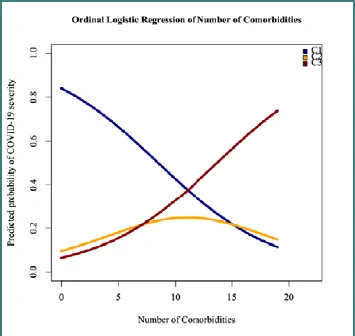
Predicted probabilities of COVID-19 severity categories stratified by the number of comorbidities. C1, mild; C2, moderate; C3, severe/critical.

When individual comorbidities were analyzed, significant associations with COVID-19 severity were identified for arterial hypertension, diabetes mellitus, thyroid disorders, and malignancies.

Notably, patients without a diagnosis of arterial hypertension (OR = 0.32; 95% CI, 0.18–0.56; *P* < 0.001), diabetes mellitus (OR = 0.25; 95% CI, 0.11–0.55; *P* = 0.001), thyroid dysfunction (OR = 0.45; 95% CI, 0.24–0.85; *P* = 0.015), and malignancy (OR = 0.37; 95% CI, 0.15–0.93; *P* = 0.035) had lower odds of developing C3 disease type. Regarding other comorbidities (e.g., obesity, chronic pulmonary disease, osteoporosis, or neurological disorders), no statistically significant associations were identified. Further details can be found in SM3 Tables S18–S25.

### Multivariable analysis of IMIRD subtype and COVID-19 severity

Median age differed across IMIRD subgroups: 63 years (interquartile range [IQR] 56–67) in patients with RA, 46 years (IQR 34–63) in those with SLE, 46 years (IQR 39–54) in those with axSpA, and 53 years (IQR 48–63) in those with SSc. Given these differences, we fitted a multivariable ordinal logistic regression model including age and IMIRD subtype, with RA as the reference category (Table 11). Increasing age was associated with greater COVID-19 severity (OR = 1.06; 95% CI, 1.03–1.09; *P* < 0.001).

**Table 11:** Age-adjusted associations between IMIRD subtype and COVID-19 severity in a multivariable ordinal logistic regression model

Parameter	OR	95% CI	*P*
1|2	46.40	8.10–265.83	**< 0.001**
2|3	131.61	21.84–793.04	**< 0.001**
SLE (vs RA)	0.35	0.13–0.90	**0.030**
axSpA (vs RA)	1.23	0.53–2.85	0.623
SSc (vs RA)	1.09	0.48–2.51	0.831
Age (years)	1.06	1.03–1.09	**< 0.001**
Observations (*n*)	211		
Nagelkerke *R*^2^	0.189		

Estimates are odds ratios from a multivariable ordinal logistic regression model including age and IMIRD subtype, with RA as the reference category with COVID-19 severity as the ordered outcome (C1, mild; C2, moderate; C3, severe/critical). Rows 1|2 and 2|3 are the threshold (intercept) parameters for the severity cut-points. Bold *P* values indicate statistical significance (*P* < 0.05). CI, confidence interval; *n*, number of patients contributing to the model; OR, odds ratio; axSpA, axial spondyloarthritis; IMIRD, immune-mediated inflammatory rheumatic disease; RA, rheumatoid arthritis; SLE, systemic lupus erythematosus; SSc, systemic sclerosis.

After adjustment for age, SLE was associated with lower odds of belonging to a greater COVID-19 severity category compared with RA (OR = 0.35; 95% CI, 0.13–0.90; *P* = 0.030). No statistically significant associations were identified for axSpA (OR = 1.23; 95% CI, 0.53–2.85; *P* = 0.623) or SSc (OR = 1.09; 95% CI, 0.48–2.51; *P* = 0.831), relative to RA, as seen in Figure 12. However, the wide confidence intervals indicate limited precision and do not establish equivalence or absence of an association (Table 11), so residual confounding cannot be excluded.

**Figure 12:**
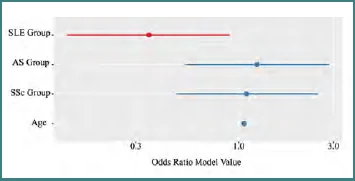
Age-adjusted associations between IMIRD subtype and COVID-19 severity estimated using a multivariable ordinal logistic regression model. Points are odds ratios and horizontal bars the 95% confidence intervals, with RA as the reference category. axSpA, axial spondyloarthritis; IMIRD, immune-mediated inflammatory rheumatic disease; OR, odds ratio; RA, rheumatoid arthritis; SLE, systemic lupus erythematosus; SSc, systemic sclerosis.

When the proposed clinical covariates and laboratory variables were entered simultaneously, the resulting ordinal logistic regression model could not be estimated reliably because of the limited complete-case sample relative to the number of model parameters. The model produced unstable or non-estimable coefficients and was therefore not retained.

## Discussion

This retrospective multicenter study examined clinical, demographic, and paraclinical determinants of COVID-19 severity in a cohort of 211 patients with IMIRD, including RA, SLE, SSc, and axSpA, a representative cohort from Romania. A key methodological advantage was restricting all laboratory data to the first 72 hours after symptom onset to minimize bias from disease progression or treatment. The analysis recognized age, baseline SpO_2_, early inflammatory markers, monocyte count, dNLR, and cumulative comorbidity burden as the main predictors of infection severity. After adjustment for age, SLE was associated with lower odds of greater COVID-19 severity compared with RA, whereas no significant associations were observed for the other IMIRD subtypes. These findings bring host-related vulnerability and acute inflammatory response to the forefront of risk stratification in this population. This work extends a previously published comparative analysis [27] in which the IMIRD cohort was compared with a non-IMIRD control group; therefore, the current study focuses on intra-cohort predictors of severity, using an ordinal outcome model that captures the full clinical spectrum rather than binary endpoints alone. This approach was intended to explore associations across severity categories, not to develop or validate a clinical prediction model.

Age was the most consistent and robust predictor of COVID-19 severity. For every year of life, the odds of greater COVID-19 severity increased by 6%, independently of IMIRD subtype. Additionally, the age-related gradient in predicted probability curves showed a particularly sharp transition to severe outcomes beyond the sixth decade of life. This pattern is well established in the broader COVID-19 literature and reflects the combined effects of immunosenescence, chronic inflammation, endothelial and metabolic dysfunction, and the progressive accumulation of age-related comorbidities [29–33].

In our analysis, baseline SpO_2_ at presentation was strongly associated with greater COVID-19 severity, and the predicted probability curves changed steeply within the low-90% range. The results confirm that baseline hypoxemia can serve as a surrogate for lung disease at presentation – a marker of vulnerability that is especially significant in the IMIRD population, who carry a substantially higher burden of pulmonary comorbidities compared to the general population [34–36]. Similar continuous patterns were observed for acute-phase reactants and hematological indices. The intersections of these curves indicate values at which the predicted probabilities of two severity categories become equal, reflecting changes in their relative distribution rather than established clinical thresholds. For laboratory biomarkers, approximate intersection values were retained only when the corresponding association remained significant after Bonferroni correction. In view of the limited evidence available from Eastern Europe for this population at increased risk of adverse COVID-19 outcomes, these model-based observations may nevertheless provide useful descriptive information and a basis for further investigation. As the values were not derived through a dedicated cut-point analysis or externally validated, they should be regarded as exploratory and hypothesis-generating.

Among laboratory parameters, acute-phase reactants – CRP, ESR, and fibrinogen – exhibited a strong graded relationship with the severity of COVID-19. The overlap of the probability curves at certain levels reflects a zone where the risk of severe SARS-CoV-2 starts to increase. These relationships are important in a population with an already existing background of inflammatory noise. Our associations are supported by an extensive body of literature from the general population [37–40] and from some populations with IMIRD [41,42]. Nonetheless, these thresholds should be interpreted as descriptive and hypothesis-generating rather than definitive clinical cut-offs.

In the unadjusted analyses, lower lymphocyte, monocyte, and eosinophil counts were associated with greater disease severity. After Bonferroni correction, however, only the association with monocyte count remained statistically significant. The nominal association with lymphopenia is nevertheless biologically plausible and consistent with previous studies in which lymphocyte depletion was frequently observed in severe or fatal COVID-19 [43–45]. However, its interpretation in patients with IMIRD requires particular caution, as lymphocyte counts may also be influenced by the underlying disease and immunomodulatory treatment.

The associations observed for monocyte depletion add further support to the concept of broader immune dysregulation as a feature of severe COVID-19 in this population.

Among the composite markers evaluated, both NLR and dNLR showed graded associations with disease severity in the unadjusted analyses. After Bonferroni correction, the association remained statistically significant for dNLR (adjusted *P* = 0.040), but not for NLR (adjusted *P* = 0.120). NLR integrates neutrophil predominance, reflecting innate immune activation, with lymphocyte depletion, commonly associated with impaired adaptive immune responses in severe SARS-CoV-2 infection [15,19,46]. Whereas NLR uses the lymphocyte count alone as its denominator, dNLR relates the neutrophil count to the entire non-neutrophil leukocyte compartment. It may therefore reflect neutrophil predominance together with reductions in several circulating leukocyte populations. This broader denominator may be relevant in patients with IMIRD, whose lymphocyte counts can already be affected by the underlying disease or immunomodulatory treatment [47,48]. Literature also supports baseline NLR elevation in patients with active RA, SSc, and axSpA, regardless of SARS-CoV-2 infection [49–54]. Another important point is that neutrophil activation, including excessive Neutrophil Extracellular Traps (NET) formation, has also been implicated in the inflammatory and thrombotic manifestations of severe COVID-19 [55].

Nevertheless, NLR and dNLR are closely related measures, and the difference observed in the present analysis should not be interpreted as evidence that dNLR is intrinsically superior.

Evidence from general COVID-19 cohorts has consistently linked elevated NLR values to severe disease, intensive care unit admission, invasive ventilation, and mortality [19,56–58]. Similar associations have been reported for dNLR, including disease progression, adverse in-hospital outcomes, and mortality, in different pandemic periods [18,46,59,60]. However, data concerning these indices in autoimmune populations remain limited. Chen *et al*. reported higher NLR values in patients with autoimmune diseases who developed severe rather than non-severe COVID-19; however, their cohort included a broader range of autoimmune conditions, and dNLR was not evaluated [61]. Our previous comparative analysis described NLR and dNLR values in patients with and without IMIRD but did not examine their relationship with severity within the IMIRD cohort [27]. To our knowledge, published evidence specifically addressing early dNLR values across the COVID-19 severity spectrum in patients with IMIRD remains scarce. Our findings suggest that the combination of neutrophilia and lymphocyte depletion captured by dNLR may reflect acute inflammatory imbalance despite the pre-existing immune abnormalities associated with IMIRDs, but requires confirmation in larger cohorts with adjustment for disease activity, immunomodulatory treatment, and other potential confounders.

These associations should be interpreted in the context of the variable and severity-dependent availability of laboratory measurements. They describe patients with available early measurements and may not fully represent the entire IMIRD cohort.

In relation to other factors associated with SARS-CoV-2 severity, the apparent protective association of vaccination requires careful interpretation. The cohort spanned the early pre-vaccination phase and later pandemic periods, during which circulating variants, population immunity, clinical management, and healthcare system preparedness changed substantially. Vaccination status was therefore intrinsically linked to calendar time, and the observed association cannot be distinguished from these pandemic-era effects. Moreover, vaccination was recorded only as a binary variable, without information on vaccine type, number of doses, or time since vaccination. The model had limited explanatory performance and did not account for calendar period, circulating viral variants, access to healthcare, or differences in health-seeking behavior. Residual confounding is therefore likely, and the association should not be interpreted as an independent or causal protective effect of vaccination.

In our cohort, the total number of comorbidities was a strong predictor of COVID-19 severity. The odds of greater COVID-19 severity increased by approximately 22% for each additional comorbid disease. Arterial hypertension, diabetes, thyroid diseases, and neoplasms proved to be among the strongest predictors of higher severity. The dose-dependent relationship observed in our sample mirrors findings from meta-analyses in larger COVID-19 populations, where cardiovascular disease and diabetes mellitus are known to indicate higher severity and have pooled ORs of approximately 2 to 4, depending on study design [62–65].

Finally, after adjustment for age, SLE was associated with lower odds of greater COVID-19 severity compared with RA. This finding should not be interpreted as evidence of an intrinsic protective effect of SLE. The differences in age distribution among the IMIRD subgroups justified adjustment for age; nevertheless, residual confounding cannot be excluded. The model did not include background antirheumatic treatment (including hydroxychloroquine exposure), disease activity, differences in comorbidity profiles, frequency of clinical monitoring, or time to presentation, which may have contributed to the observed association. Moreover, the relatively small subgroup sizes and wide confidence intervals limited the precision of the estimates. We did not identify statistically significant associations for axSpA or SSc relative to RA, but these findings do not demonstrate equivalence or exclude clinically relevant associations. Specifically, patients with SLE in this cohort were, on average, younger than those with RA (46 vs. 63 years old) – a difference reflecting the well-established epidemiological distinction between these two diseases. RA most commonly presents in the fourth to sixth decade of life [66,67], whereas SLE typically has its onset in the second to fourth decade, with peak incidence during the reproductive years [68,69]. This age difference likely contributed to the lower unadjusted severity observed in SLE, although age was included in the adjusted model. An age-matched subgroup analysis was not undertaken because matching would have further reduced the already limited subgroup samples and the precision of the estimates. Confirmation in larger cohorts permitting adequately powered age-matched analyses is therefore warranted. Baseline hydroxychloroquine use was recorded in 82.2% of patients with SLE and 13.3% of those with RA (Supplementary Material 1). This difference may represent a source of residual confounding; however, the present study was not designed to determine whether hydroxychloroquine contributed to the lower adjusted severity observed in the SLE group. Treatment duration, adherence, and its continuation during the acute infection could not be established consistently, and the independent effect of this therapy could not be properly assessed. Although hydroxychloroquine was initially investigated for its potential antiviral and immunomodulatory effects [70–72], subsequent randomized trials did not demonstrate a clinical benefit in patients hospitalized with COVID-19 [73,74]. More favorable signals were reported for some other repurposed antiviral agents. In the case of favipiravir, several studies reported reductions in viral load, improvement in clinical symptoms, and shorter hospital stays [75,76]. In contrast, patients with RA were older and carried a higher burden of cardiovascular and metabolic comorbidities, factors that independently increase the risk of severe COVID-19. It is also possible that the typically closer clinical monitoring of patients with SLE – a consequence of more frequent rheumatology follow-up for a disease with greater systemic complexity – led to earlier recognition and a more timely clinical presentation, thus possibly enabling an earlier therapeutic approach. In this context, RA may represent a more vulnerable comparator rather than a neutral reference.

On the other hand, the use of separate predictor-specific models does not permit assessment of the independent contribution of each clinical and laboratory variable. Although a more extensively adjusted ordinal regression model was explored, the reduced number of complete observations, the limited size of the higher severity categories, and the overlap among inflammatory biomarkers prevented reliable estimation of all coefficients within a single model. Residual confounding therefore remains possible, and the reported associations should not be interpreted as independent predictive effects.

### Strengths and limitations

This study has several strengths. It was conducted in a multicenter cohort of patients with IMIRD and included a comprehensive assessment of demographic, clinical, and laboratory factors, including some composite indices associated with COVID-19 severity. Laboratory parameters were collected within the first 72 hours following SARS-CoV-2 symptom onset, providing insight into early inflammatory changes during the acute phase of infection. In contrast to many previous studies that classified the infection simply as severe or non-severe, we evaluated outcomes across the entire spectrum of COVID-19 severity, allowing a more detailed assessment of factors associated with disease progression. SARS-CoV-2 severity was established using objective clinical data collected from medical records, reducing the risk of misclassification. Furthermore, the analysis included an adjusted comparison between IMIRD subtypes, making it possible to explore disease-specific differences while accounting for the effect of age.

Several limitations that are also inherent to the retrospective observational design should be acknowledged. Laboratory tests were not obtained uniformly, as their availability depended on disease severity, clinical indication, care setting, and local practice. Although on the one hand a strength of the study, on the other, restricting the analysis to measurements obtained during the first 72 hours further reduced data availability. Consequently, some biomarkers were insufficiently available for analysis, while missing observations were more frequent in C1. The paraclinical findings should therefore be considered exploratory and require confirmation in prospective cohorts using standardized laboratory assessment.

No a priori sample size calculation was performed because all eligible patients were included; however, the overall sample size and smaller complete-case samples for several biomarkers limited statistical precision and the detection of modest associations.

Additionally, vaccination information was captured in a binary (yes/no) manner, without further details, limiting a thorough assessment of this variable (however, as mentioned above, this was not our main aim). Consequently, the observed protective effect of vaccination cannot be distinguished from era effects, as unvaccinated patients were predominantly infected during the pre-vaccination period when more virulent strains circulated, or from differences in health-seeking behavior.

Antirheumatic and immunomodulatory treatment differed across IMIRD subtypes, and the available records did not consistently document treatment duration, adherence, or continuation during acute infection. It was therefore not possible to distinguish treatment effects from those related to disease subtype, disease activity, or the indication for therapy. Any association with severity would therefore be particularly susceptible to confounding by indication and reverse causality.

Finally, the proportional odds assumption was not formally evaluated using a dedicated statistical test. Although models allowing non-proportional effects were explored, they did not achieve reliable convergence. The findings derived from the ordinal regression analyses should therefore be interpreted with appropriate caution and reassessed in larger prospective cohorts with standardized laboratory testing.

## Conclusion

In the present Romanian cohort, COVID-19 severity in patients with IMIRD was more closely related to host factors – age, systemic inflammation, and overall comorbidity burden – than to the underlying IMIRD diagnosis. Across disease subtypes, the differences were limited; SLE was associated with lower odds of belonging to a higher severity category. Because a single multivariable model incorporating all relevant clinical and laboratory variables could not be reliably estimated, these associations should not be interpreted as independent effects.

In practice, these findings support the idea of early risk assessment based on age, comorbidity burden, and available laboratory parameters, including ESR, CRP, fibrinogen, dNLR, and monocyte counts. Identifying higher-risk patients early may help guide monitoring and care. These findings should be interpreted within the above-mentioned limits of the present study. Further research in larger, prospective cohorts is needed to clarify how disease activity, background immunomodulatory therapy, and circulating viral variants interact, including any potential links between vaccination status and variant selection.

### Key findings

- In 211 patients with IMIRD and COVID-19, greater severity was associated with older age and with a higher number of comorbidities.
- Early acute-phase reactants (ESR, CRP, fibrinogen), a higher dNLR, and a lower monocyte count remained associated with severity after Bonferroni correction.
- After adjustment for age, SLE was associated with lower odds of greater severity than RA, whereas no differences were observed for axSpA or SSc.

### Implications for practice

- Routine, inexpensive parameters obtained within the first 72 hours of symptom onset may support early risk stratification in patients with IMIRD.
- The curve intersections reported here are descriptive and should not be used as clinical decision thresholds until they are validated.
- Assessing COVID-19 severity across ordered categories may provide a more informative picture of clinical progression than a simple severe/non-severe classification.

## Data Availability

Further data are available from the corresponding author upon reasonable request.

## References

[1] Grainger R, Kim AHJ, Conway R, Yazdany J, Robinson PC (2022). COVID-19 in people with rheumatic diseases: risks, outcomes, treatment considerations. Nat Rev Rheumatol.

[2] Conway R, Grimshaw AA, Konig MF, Putman M, Duarte-García A, Tseng LY (2022). SARS-CoV-2 infection and COVID-19 outcomes in rheumatic diseases: a systematic literature review and meta-analysis. Arthritis Rheumatol.

[3] Grainger R, Machado PM, Robinson PC (2021). Novel coronavirus disease-2019 (COVID-19) in people with rheumatic disease: epidemiology and outcomes. Best Pract Res Clin Rheumatol.

[4] Svensson ALL, Emborg HD, Bartels LE, Ellingsen T, Adelsten T, Cordtz R (2023). Outcomes following SARS-CoV-2 infection in individuals with and without inflammatory rheumatic diseases: a Danish nationwide cohort study. Ann Rheum Dis.

[5] MacKenna B, Kennedy NA, Mehrkar A, Rowan A, Galloway J, Matthewman J (2022). Risk of severe COVID-19 outcomes associated with immune-mediated inflammatory diseases and immune-modifying therapies: a nationwide cohort study in the OpenSAFELY platform. Lancet Rheumatol.

[6] Saadoun D, Vieira M, Vautier M, Baraliakos X, Andreica I, da Silva JAP (2021). SARS-CoV-2 outbreak in immune-mediated inflammatory diseases: the Euro-COVIMID multicentre cross-sectional study. Lancet Rheumatol.

[7] Boteanu A, Leon L, Esteban SP, Rubio ER, Pascual MP, Bonilla G (2023). Severe COVID-19 in patients with immune-mediated rheumatic diseases: a stratified analysis from the SORCOM multicentre registry. Mod Rheumatol.

[8] Raiker R, DeYoung C, Pakhchanian H, Ahmed S, Kavadichanda C, Gupta L (2021). Outcomes of COVID-19 in patients with rheumatoid arthritis: a multicenter research network study in the United States. Semin Arthritis Rheum.

[9] Kwiatkowska B, Krajewska-Włodarczyk M, Batko B, Maślińska M, Stajszczyk M, Świerkot J (2024). COVID-19 prophylaxis, diagnostics, and treatment in patients with rheumatic diseases. The Polish experts panel opinion. Reumatologia.

[10] Rorat M, Zarębska-Michaluk D, Kowalska J, Kujawa K, Rogalska M, Kozielewicz D (2022). The course of COVID-19 in patients with systemic autoimmune rheumatic diseases. J Clin Med.

[11] Pinte L, Caraiola S, Balaban DV, Badea C, Mazilu D, Ionescu GD (2021). COVID-19 impact and vaccination willingness among Romanian patients with autoimmune/immune-mediated diseases. Healthcare (Basel).

[12] Vlădulescu-Trandafir AI, Bojincă VC, Popescu C, Munteanu C, Bălănescu AR, Anghelescu A (2025). Predictive factors for COVID-19 severity in patients with axial spondyloarthritis: real-world data from the Romanian Registry of Rheumatic Diseases. Medicina (Kaunas).

[13] Strangfeld A, Schäfer M, Gianfrancesco MA, Lawson-Tovey S, Liew JW, Ljung L (2021). Factors associated with COVID-19-related death in people with rheumatic diseases: results from the COVID-19 Global Rheumatology Alliance physician-reported registry. Ann Rheum Dis.

[14] Wang Q, Liu J, Shao R, Han X, Su C, Lu W (2021). Risk and clinical outcomes of COVID-19 in patients with rheumatic diseases compared with the general population: a systematic review and meta-analysis. Rheumatol Int.

[15] Li J, Zhang K, Zhang Y, Gu Z, Huang C (2023). Neutrophils in COVID-19: recent insights and advances. Virol J.

[16] An H, Zhang J, Li T, Hu Y, Wang Q, Chen C (2022). Inflammation/coagulopathy/immunology responsive index predicts poor COVID-19 prognosis. Front Cell Infect Microbiol.

[17] Semiz S (2022). COVID-19 biomarkers: what did we learn from systematic reviews?. Front Cell Infect Microbiol.

[18] Qiu W, Shi Q, Chen F, Wu Q, Yu X, Xiong L (2022). The derived neutrophil to lymphocyte ratio can be the predictor of prognosis for COVID-19 Omicron BA.2 infected patients. Front Immunol.

[19] Li X, Liu C, Mao Z, Xiao M, Wang L, Qi S (2020). Predictive values of neutrophil-to-lymphocyte ratio on disease severity and mortality in COVID-19 patients: a systematic review and meta-analysis. Crit Care.

[20] Tang F, Zhang X, Zhang B, Zhu B, Wang J (2022). A nomogram prediction of outcome in patients with COVID-19 based on individual characteristics incorporating immune response-related indicators. J Med Virol.

[21] Zinellu A, Paliogiannis P, Mangoni AA (2023). Aggregate index of systemic inflammation (AISI), disease severity, and mortality in COVID-19: a systematic review and meta-analysis. J Clin Med.

[22] Gianfrancesco M, Hyrich KL, Al-Adely S, Carmona L, Danila MI, Gossec L (2020). Characteristics associated with hospitalisation for COVID-19 in people with rheumatic disease: data from the COVID-19 Global Rheumatology Alliance physician-reported registry. Ann Rheum Dis.

[23] Wang F, Ma Y, Xu S, Liu H, Chen Y, Yang H (2022). Prevalence and risk of COVID-19 in patients with rheumatic diseases: a systematic review and meta-analysis. Clin Rheumatol.

[24] Bournia VK, Fragoulis GE, Mitrou P, Mathioudakis K, Tsolakidis A, Konstantonis G (2023). Different COVID-19 outcomes among systemic rheumatic diseases: a nation-wide cohort study. Rheumatology (Oxford).

[25] Zhai J, Smith F, Soon G (2025). Comparison of continuous, binary, and ordinal endpoints. J Biopharm Stat.

[26] Verhulst B, Neale MC (2021). Best Practices for Binary and Ordinal Data Analyses. Behav Genet.

[27] Vlădulescu-Trandafir AI, Onose G, Bojincă VC, Mirea A, Aurelian SM, Munteanu C (2026). Mapping early COVID-19 clinical and laboratory profiles in patients with immune-mediated inflammatory rheumatic diseases: a multicenter case-control study. Balneo PRM Res J.

[28] R Core Team (2024). R: a language and environment for statistical computing. [Internet].

[29] Farshbafnadi M, Kamali Zonouzi S, Sabahi M, Dolatshahi M, Aarabi MH (2021). Aging & COVID-19 susceptibility, disease severity, and clinical outcomes: the role of entangled risk factors. Exp Gerontol.

[30] Starke KR, Reissig D, Petereit-Haack G, Schmauder S, Nienhaus A, Seidler A (2021). The isolated effect of age on the risk of COVID-19 severe outcomes: a systematic review with meta-analysis. BMJ Glob Health.

[31] Ebrahimzadeh A, Mohseni S, Narimani B, Ebrahimzadeh A, Kazemi S, Keshavarz F (2023). Association between vitamin D status and risk of COVID-19 in-hospital mortality: a systematic review and meta-analysis of observational studies. Crit Rev Food Sci Nutr.

[32] Vuille-dit-Bille RN, Liechty KW, Verrey F, Guglielmetti LC (2020). SARS-CoV-2 receptor ACE2 gene expression in small intestine correlates with age. Amino Acids.

[33] Baker SA, Kwok S, Berry GJ, Montine TJ (2021). Angiotensin-converting enzyme 2 (ACE2) expression increases with age in patients requiring mechanical ventilation. PLoS One.

[34] Huang H, Zhang M, Chen C, Zhang H, Wei Y, Tian J (2020). Clinical characteristics of COVID-19 in patients with preexisting ILD: a retrospective study in a single center in Wuhan, China. J Med Virol.

[35] Lee H, Choi H, Yang B, Lee SK, Park TS, Park DW (2021). Interstitial lung disease increases susceptibility to and severity of COVID-19. Eur Respir J.

[36] Joy GM, Arbiv OA, Wong CK, Lok SD, Adderley NA, Dobosz KM (2023). Prevalence, imaging patterns and risk factors of interstitial lung disease in connective tissue disease: a systematic review and meta-analysis. Eur Respir Rev.

[37] De Rop L, Bos DAG, Stegeman I, Holtman G, Ochodo EA, Spijker R (2024). Accuracy of routine laboratory tests to predict mortality and deterioration to severe or critical COVID-19 in people with SARS-CoV-2. Cochrane Database Syst Rev.

[38] Wang L, Yang LM, Pei SF, Chong YZ, Guo Y, Gao XL (2021). CRP, SAA, LDH, and DD predict poor prognosis of coronavirus disease (COVID-19): a meta-analysis from 7739 patients. Scand J Clin Lab Invest.

[39] Ponti G, Maccaferri M, Ruini C, Tomasi A, Ozben T (2020). Biomarkers associated with COVID-19 disease progression. Crit Rev Clin Lab Sci.

[40] Hariyanto TI, Japar KV, Kwenandar F, Damay V, Siregar JI, Lugito NPH, Tjiang MM, Kurniawan A (2021). Inflammatory and hematologic markers as predictors of severe outcomes in COVID-19 infection: A systematic review and meta-analysis. Am J Emerg Med.

[41] Santos CS, Morales CM, Álvarez ED, Castro CÁ, Robles AL, Sandoval TP (2020). Determinants of COVID-19 disease severity in patients with underlying rheumatic disease. Clin Rheumatol.

[42] Hsu TYT, D’Silva KM, Patel NJ, Wang J, Mueller AA, Fu X (2021). Laboratory trends, hyperinflammation, and clinical outcomes for patients with a systemic rheumatic disease admitted to hospital for COVID-19: a retrospective, comparative cohort study. Lancet Rheumatol.

[43] Henry BM, Cheruiyot I, Vikse J, Mutua V, Kipkorir V, Benoit J (2020). Lymphopenia and neutrophilia at admission predicts severity and mortality in patients with COVID-19: a meta-analysis. Acta Biomed.

[44] Terpos E, Ntanasis-Stathopoulos I, Elalamy I, Kastritis E, Sergentanis TN, Politou M (2020). Hematological findings and complications of COVID-19. Am J Hematol.

[45] Fan BE, Chong VCL, Chan SSW, Lim GH, Lim KGE, Tan GB (2020). Hematologic parameters in patients with COVID-19 infection. Am J Hematol.

[46] Citu C, Gorun F, Motoc A, Sas I, Gorun OM, Burlea B (2022). The predictive role of NLR, dNLR, MLR, and SIRI in COVID-19 mortality. Diagnostics (Basel).

[47] Moysidou E, Christodoulou M, Lioulios G, Stai S, Karamitsos T, Dimitroulas T (2024). Lymphocytes change their phenotype and function in systemic lupus erythematosus and lupus nephritis. Int J Mol Sci.

[48] Wu YX, Zhang W, Liao Y, Sun T, Liu Y, Liu Y (2025). Immune cell aberrations in systemic lupus erythematosus: navigating the targeted therapies toward precision management. Cell Mol Biol Lett.

[49] Yang X (2025). The supplementary value of the neutrophil-to-lymphocyte ratio in the diagnosis of rheumatoid arthritis. Medicine (Baltimore).

[50] Li Y, Liu J, Hu Y, Cong C, Chen Y, Fang Y (2025). The neutrophil-to-lymphocyte ratio in rheumatoid arthritis: the dual perspectives from literature and clinic. Medicine (Baltimore).

[51] Almirall M, Espartal E, Michelena X, Suso-Ribera C, Serrat M, Marsal S (2025). Neutrophil–lymphocyte ratio in fibromyalgia and axial spondyloarthritis: a potential biomarker for diagnosis and disease activity. Biomedicines.

[52] Mercan R, Bitik B, Tufan A, Bozbulut UB, Atas N, Ozturk MA (2016). The association between neutrophil/lymphocyte ratio and disease activity in rheumatoid arthritis and ankylosing spondylitis. J Clin Lab Anal.

[53] Zinellu A, Mangoni AA (2023). Neutrophil-to-lymphocyte and platelet-to-lymphocyte ratio and disease activity in rheumatoid arthritis: a systematic review and meta-analysis. Eur J Clin Invest.

[54] Zinellu A, Mangoni AA (2024). The association between the neutrophil-to-lymphocyte ratio, platelet-to-lymphocyte ratio, and monocyte-to-lymphocyte ratio and systemic sclerosis and its complications: a systematic review and meta-analysis. Front Immunol.

[55] Al-Kuraishy HM, Al-Gareeb AI, Al-hussaniy HA, Al-Harcan NAH, Alexiou A, Batiha GES (2022). Neutrophil extracellular traps (NETs) and COVID-19: a new frontiers for therapeutic modality. Int Immunopharmacol.

[56] Yildiz H, Castanares-Zapatero D, Pierman G, Pothen L, De Greef J, Nana FA (2021). Validation of neutrophil-to-lymphocyte ratio cut-off value associated with high in-hospital mortality in COVID-19 patients. Int J Gen Med.

[57] Regolo M, Vaccaro M, Sorce A, Stancanelli B, Colaci M, Natoli G (2022). Neutrophil-to-lymphocyte ratio (NLR) is a promising predictor of mortality and admission to intensive care unit of COVID-19 patients. J Clin Med.

[58] Alonso Batun AI, Tello Romero VJ, Pacheco Polanco OA, Mendez-Dominguez N (2025). Neutrophil-to-lymphocyte ratio predicts COVID-19 mortality in the Yucatecan Maya population: a retrospective cohort study. COVID.

[59] Ardestani SK, Salehi MR, Attaran B, Hashemi SM, Sadeghi S, Ghaffarpour S (2022). Neutrophil to lymphocyte ratio (NLR) and derived NLR combination: a cost-effective predictor of moderate to severe COVID-19 progression. Iran J Allergy Asthma Immunol.

[60] Ramesh J, Reddy SLS, Rajesh M, Varghese J (2021). Evaluation of simple and cost-effective immunohaematological markers to predict outcome in hospitalized severe COVID-19 patients, with a focus on diabetes mellitus-A retrospective study in Andhra Pradesh, India. Diabetes Metab Syndr.

[61] Chen LM, Li JB, Wu R (2024). Predictors of COVID-19 severity in autoimmune disease patients: a retrospective study during full epidemic decontrol in China. Heart Lung.

[62] Booth A, Reed AB, Ponzo S, Yassaee A, Aral M, Plans D (2021). Population risk factors for severe disease and mortality in COVID-19: a global systematic review and meta-analysis. PLoS One.

[63] Di Castelnuovo A, Bonaccio M, Costanzo S, Gialluisi A, Antinori A, Berselli N (2020). Common cardiovascular risk factors and in-hospital mortality in 3,894 patients with COVID-19: survival analysis and machine learning-based findings from the multicentre Italian CORIST study. Nutr Metab Cardiovasc Dis.

[64] Mehta P, Gasparyan AY, Zimba O, Kitas GD, Yessirkepov M (2022). Interplay of diabetes mellitus and rheumatic diseases amidst the COVID-19 pandemic: influence on the risk of infection, outcomes, and immune responses. Clin Rheumatol.

[65] Ahmed S, Gasparyan AY, Zimba O (2021). Comorbidities in rheumatic diseases need special consideration during the COVID-19 pandemic. Rheumatol Int.

[66] Bergstra SA, Sepriano A, Chopra A, Winchow LL, Vega-Morales D, Salomon-Escoto K (2023). Country-level socioeconomic status relates geographical latitude to the onset of RA: a worldwide cross-sectional analysis in the METEOR registry. Ann Rheum Dis.

[67] Shimizu M, Paudel ML, Shadick N, Weinblatt M, Solomon DH (2025). Age of onset of rheumatoid arthritis and radiographic changes. Semin Arthritis Rheum.

[68] Irure-Ventura J, López-Hoyos M (2022). Disease criteria of systemic lupus erythematosus (SLE); the potential role of non-criteria autoantibodies. J Transl Autoimmun.

[69] Dai X, Fan Y, Zhao X (2025). Systemic lupus erythematosus: updated insights on the pathogenesis, diagnosis, prevention and therapeutics. Signal Transduct Target Ther.

[70] Dima A, Jurcut C, Chasset F, Felten R, Arnaud L (2022). Hydroxychloroquine in systemic lupus erythematosus: overview of current knowledge. Ther Adv Musculoskelet Dis.

[71] Schilling WHK, Mukaka M, Callery JJ, Llewelyn MJ, Cruz CV, Dhorda M (2024). Evaluation of hydroxychloroquine or chloroquine for the prevention of COVID-19 (COPCOV): a double-blind, randomised, placebo-controlled trial. PLoS Med.

[72] Million M, Lagier JC, Gautret P, Colson P, Fournier PE, Amrane S (2020). Early treatment of COVID-19 patients with hydroxychloroquine and azithromycin: A retrospective analysis of 1061 cases in Marseille, France. Travel Med Infect Dis.

[73] Hong H, Friedland A, Hu M, Anstrom KJ, Halabi S, McKinnon JE (2023). Safety and efficacy of hydroxychloroquine as prophylactic against COVID-19 in healthcare workers: a meta-analysis of randomised clinical trials. BMJ Open.

[74] RECOVERY Collaborative Group (2020). Effect of hydroxychloroquine in hospitalized patients with COVID-19. N Engl J Med.

[75] Shinkai M, Tsushima K, Tanaka S, Hagiwara E, Tarumoto N, Kawada I (2021). Efficacy and safety of favipiravir in moderate COVID-19 pneumonia patients without oxygen therapy: a randomized, phase III clinical trial. Infect Dis Ther.

[76] Kumar K, Vidyadhara S, Babu J (2023). Favipiravir: emergent antiviral drug during COVID-19. Med Pharm J.

