# Supplementary material for: COVID-19 severity and early inflammatory biomarkers in patients with immune-mediated inflammatory rheumatic diseases: a multicenter cohort study
Source: J Med Life. 2026 Aug;19(8):657–71. doi: 10.25122/jml-2026-0098 (PMC13674051; doi:10.25122/jml-2026-0098)
Supplement: Supplementary file 1 [file JMedLife-19-657-s001.pdf]

Supplementary Table 1. Background antirheumatic therapy and COVID-19-directed corticosteroid use by IMIRD subtype

Treatment	Overall (*n* = 211)	RA (*n* = 90)	SLE (*n* = 45)	axSpA (*n* = 46)	SSc (*n* = 30)
**Glucocorticoid (GC) exposure**					
Background GC	68 (32.2%)	35 (38.9%)	25 (55.6%)	0 (0.0%)	8 (26.7%)
GC-dependent	64 (30.3%)	34 (37.8%)	22 (48.9%)	0 (0.0%)	8 (26.7%)
**csDMARDs and immunosuppressive agents**					
Any conventional agent	139 (65.9%)	68 (75.6%)	40 (88.9%)	11 (23.9%)	20 (66.7%)
Hydroxychloroquine	49 (23.2%)	12 (13.3%)	37 (82.2%)	0 (0.0%)	0 (0.0%)
Methotrexate	43 (20.4%)	33 (36.7%)	3 (6.7%)	2 (4.3%)	5 (16.7%)
Leflunomide	20 (9.5%)	20 (22.2%)	0 (0.0%)	0 (0.0%)	0 (0.0%)
Sulfasalazine	18 (8.5%)	9 (10.0%)	0 (0.0%)	9 (19.6%)	0 (0.0%)
Azathioprine	15 (7.1%)	2 (2.2%)	9 (20.0%)	0 (0.0%)	4 (13.3%)
Mycophenolate mofetil	16 (7.6%)	0 (0.0%)	5 (11.1%)	0 (0.0%)	11 (36.7%)
Cyclophosphamide	2 (0.9%)	0 (0.0%)	2 (4.4%)	0 (0.0%)	0 (0.0%)
**tsDMARDs**					
Any JAK inhibitor	13 (6.2%)	13 (14.4%)	0 (0.0%)	0 (0.0%)	0 (0.0%)
Tofacitinib	3 (1.4%)	3 (3.3%)	0 (0.0%)	0 (0.0%)	0 (0.0%)
Baricitinib	8 (3.8%)	8 (8.9%)	0 (0.0%)	0 (0.0%)	0 (0.0%)
Upadacitinib	2 (0.9%)	2 (2.2%)	0 (0.0%)	0 (0.0%)	0 (0.0%)
**bDMARDs**					
Any bDMARD	67 (31.8%)	29 (32.2%)	6 (13.3%)	28 (60.9%)	4 (13.3%)
TNF-α inhibitor	38 (18.0%)	11 (12.2%)	0 (0.0%)	27 (58.7%)	0 (0.0%)
IL-6 inhibitor	1 (0.5%)	1 (1.1%)	0 (0.0%)	0 (0.0%)	0 (0.0%)
IL-17 inhibitor	1 (0.5%)	0 (0.0%)	0 (0.0%)	1 (2.2%)	0 (0.0%)
IL-12/23 inhibitor	1 (0.5%)	0 (0.0%)	0 (0.0%)	1 (2.2%)	0 (0.0%)
T-cell co-stimulation modulator (abatacept)	2 (0.9%)	2 (2.2%)	0 (0.0%)	0 (0.0%)	0 (0.0%)
B-cell-targeted therapy	25 (11.8%)	15 (16.7%)	6 (13.3%)	0 (0.0%)	4 (13.3%)
Rituximab	19 (9.0%)	15 (16.7%)	0 (0.0%)	0 (0.0%)	4 (13.3%)
Belimumab	6 (2.8%)	0 (0.0%)	6 (13.3%)	0 (0.0%)	0 (0.0%)
**Treatment prescribed for acute COVID-19**					
Corticosteroids	72 (34.1%)	44 (48.9%)	8 (17.8%)	10 (21.7%)	10 (33.3%)

Data are presented as *n* (%) of patients within each column group. Patients could have more than one therapy recorded; treatment categories are not mutually exclusive, and component percentages may therefore exceed the aggregate class percentage when summed. The bDMARD group included TNF-α inhibitors, interleukin-pathway inhibitors, abatacept, rituximab, and belimumab. Corticosteroids prescribed specifically for COVID-19 were recorded separately from background glucocorticoid treatment. axSpA, axial spondyloarthritis; bDMARD, biologic disease-modifying antirheumatic drug; COVID-19, coronavirus disease 2019; csDMARD, conventional synthetic disease-modifying antirheumatic drug; DMARD, disease-modifying antirheumatic drug; GC, glucocorticoid; IL, interleukin; IMIRD, immune-mediated inflammatory rheumatic disease; JAK, Janus kinase; *n*, number of patients; RA, rheumatoid arthritis; SLE, systemic lupus erythematosus; SSc, systemic sclerosis; TNF, tumor necrosis factor; tsDMARD, targeted synthetic disease-modifying antirheumatic drug.

© 2026 by the authors. This article is an open access article distributed under the terms and conditions of the Creative Commons Attribution (CC BY 4.0) license.
