# Supplementary material for: COVID-19 severity and early inflammatory biomarkers in patients with immune-mediated inflammatory rheumatic diseases: a multicenter cohort study
Source: J Med Life. 2026 Aug;19(8):657–71. doi: 10.25122/jml-2026-0098 (PMC13674051; doi:10.25122/jml-2026-0098)
Supplement: Supplementary file 3 [file JMedLife-19-657-s003.pdf]

Table S1. Ordinal logistic regression analysis of the association between sex and COVID-19 severity

Parameter	OR	95% CI	*P*
1|2	1.79	1.30–2.46	**< 0.001**
2|3	4.44	3.04–6.49	**< 0.001**
Male (vs female)	1.03	0.54–1.96	0.930
Observations (*n*)	211		

Estimates are odds ratios from a univariable ordinal logistic regression model with COVID-19 severity as the ordered outcome (C1, mild; C2, moderate; C3, severe/critical). Rows 1|2 and 2|3 are the threshold (intercept) parameters for the severity cut-points. Bold *P* values indicate statistical significance (*P* < 0.05). CI, confidence interval; *n*, number of patients contributing to the model; OR, odds ratio.

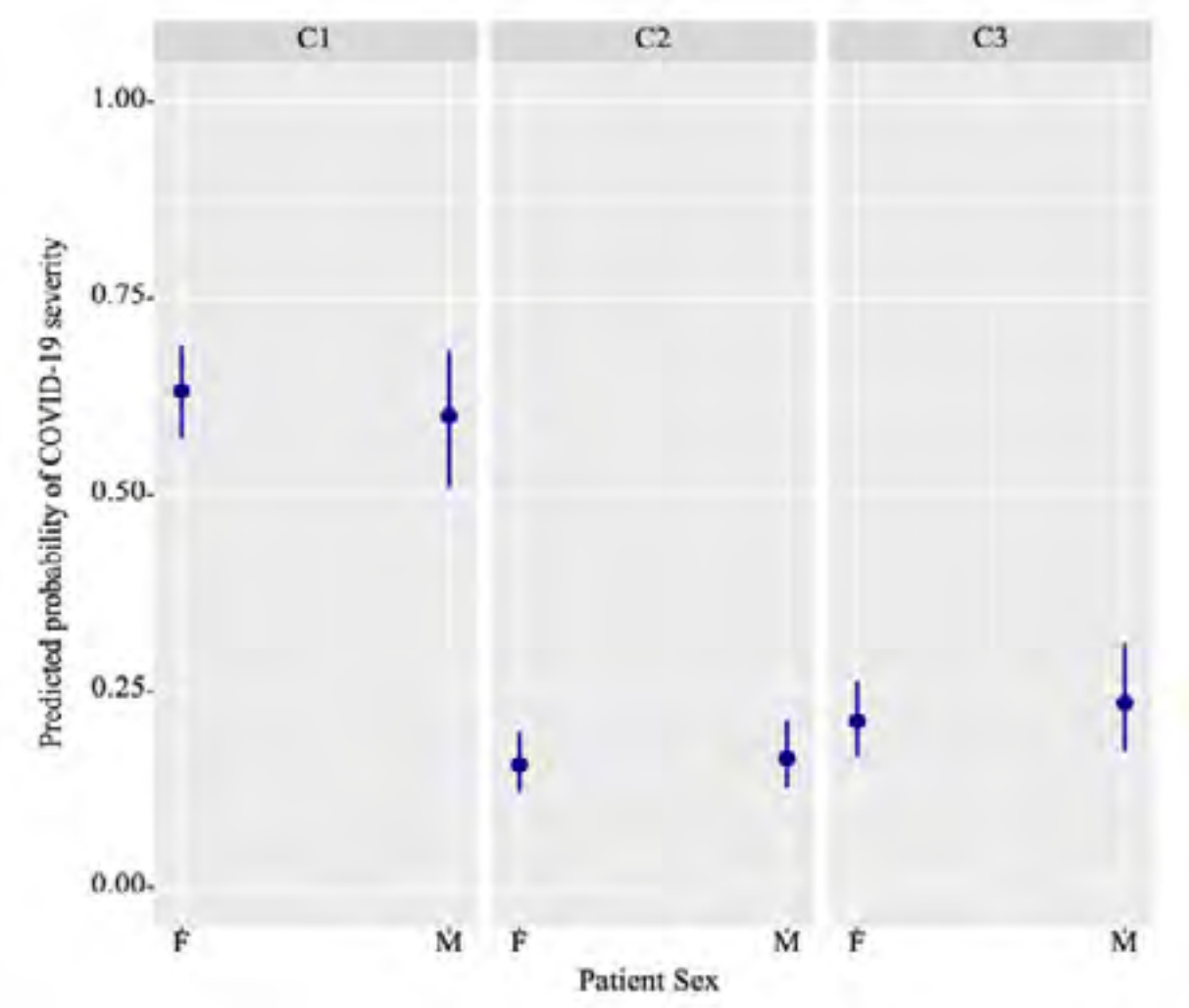

Figure S1. Predicted probabilities of COVID-19 severity categories according to patient sex. Points are predicted probabilities and vertical bars the 95% confidence intervals. C1, mild; C2, moderate; C3, severe/critical; F, female; M, male.

Table S2. Ordinal logistic regression analysis of the association between environment of origin and COVID-19 severity

Parameter	OR	95% CI	*P*
1|2	2.76	1.51–5.06	**0.001**
2|3	6.92	3.62–13.24	**< 0.001**
Urban (vs rural)	1.78	0.90–3.50	0.097
Observations (*n*)	211		

Estimates are odds ratios from a univariable ordinal logistic regression model with COVID-19 severity as the ordered outcome (C1, mild; C2, moderate; C3, severe/critical). Rows 1|2 and 2|3 are the threshold (intercept) parameters for the severity cut-points. Bold *P* values indicate statistical significance (*P* < 0.05). CI, confidence interval; *n*, number of patients contributing to the model; OR, odds ratio.

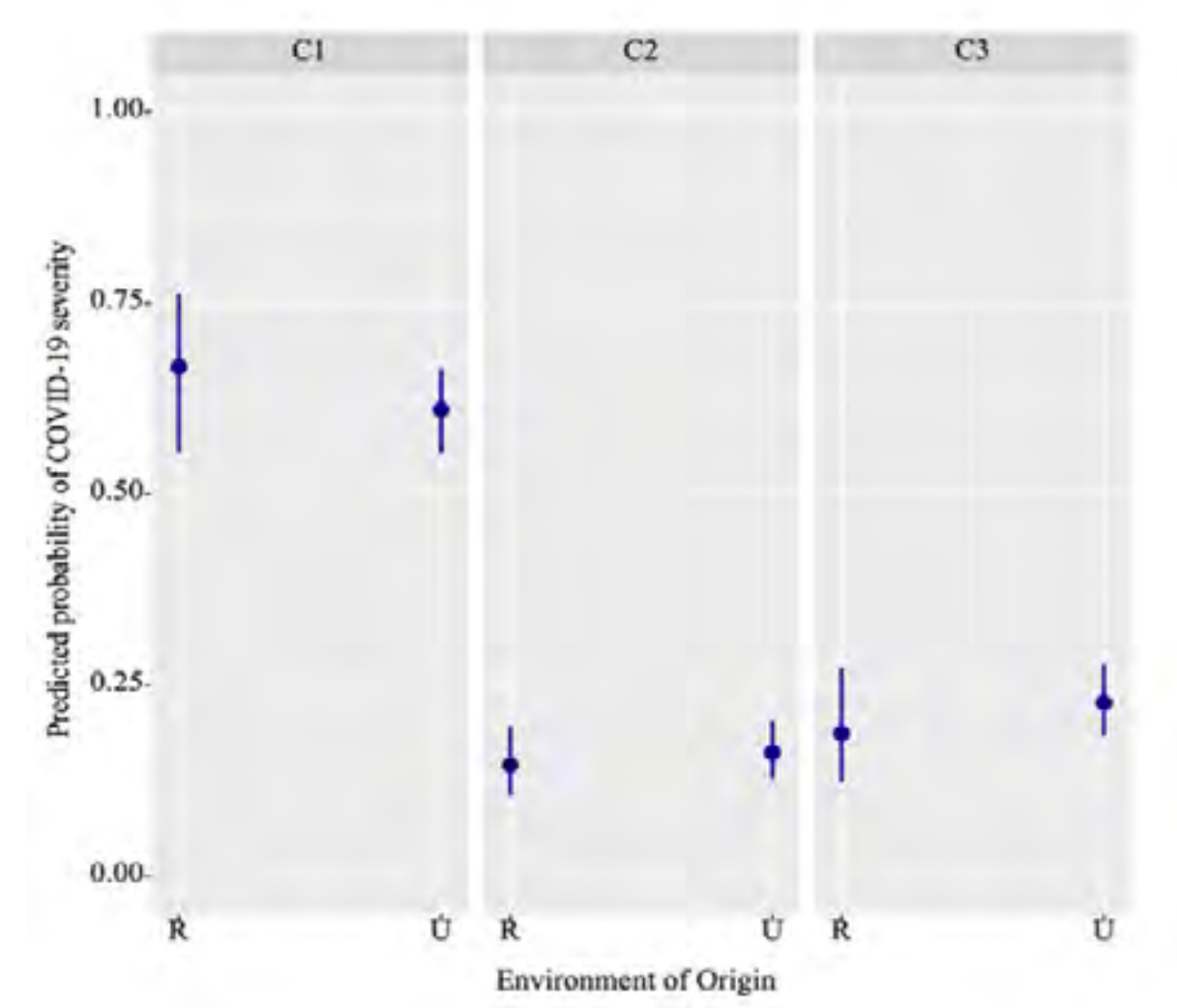

Figure S2. Model-predicted probabilities of COVID-19 severity categories according to the environment of origin. Points are predicted probabilities and vertical bars the 95% confidence intervals. C1, mild; C2, moderate; C3, severe/critical; R, rural; U, urban.

Table S3. Ordinal logistic regression analysis of the association between smoking status and COVID-19 severity

Parameter	OR	95% CI	*P*
1|2	3.46	1.29–9.30	**0.014**
2|3	8.65	3.13–23.93	**< 0.001**
Non-smoker (vs smoker)	2.09	0.75–5.84	0.160
Observations (*n*)	211		

Estimates are odds ratios from a univariable ordinal logistic regression model with COVID-19 severity as the ordered outcome (C1, mild; C2, moderate; C3, severe/critical). Rows 1|2 and 2|3 are the threshold (intercept) parameters for the severity cut-points. Bold *P* values indicate statistical significance (*P* < 0.05). CI, confidence interval; *n*, number of patients contributing to the model; OR, odds ratio.

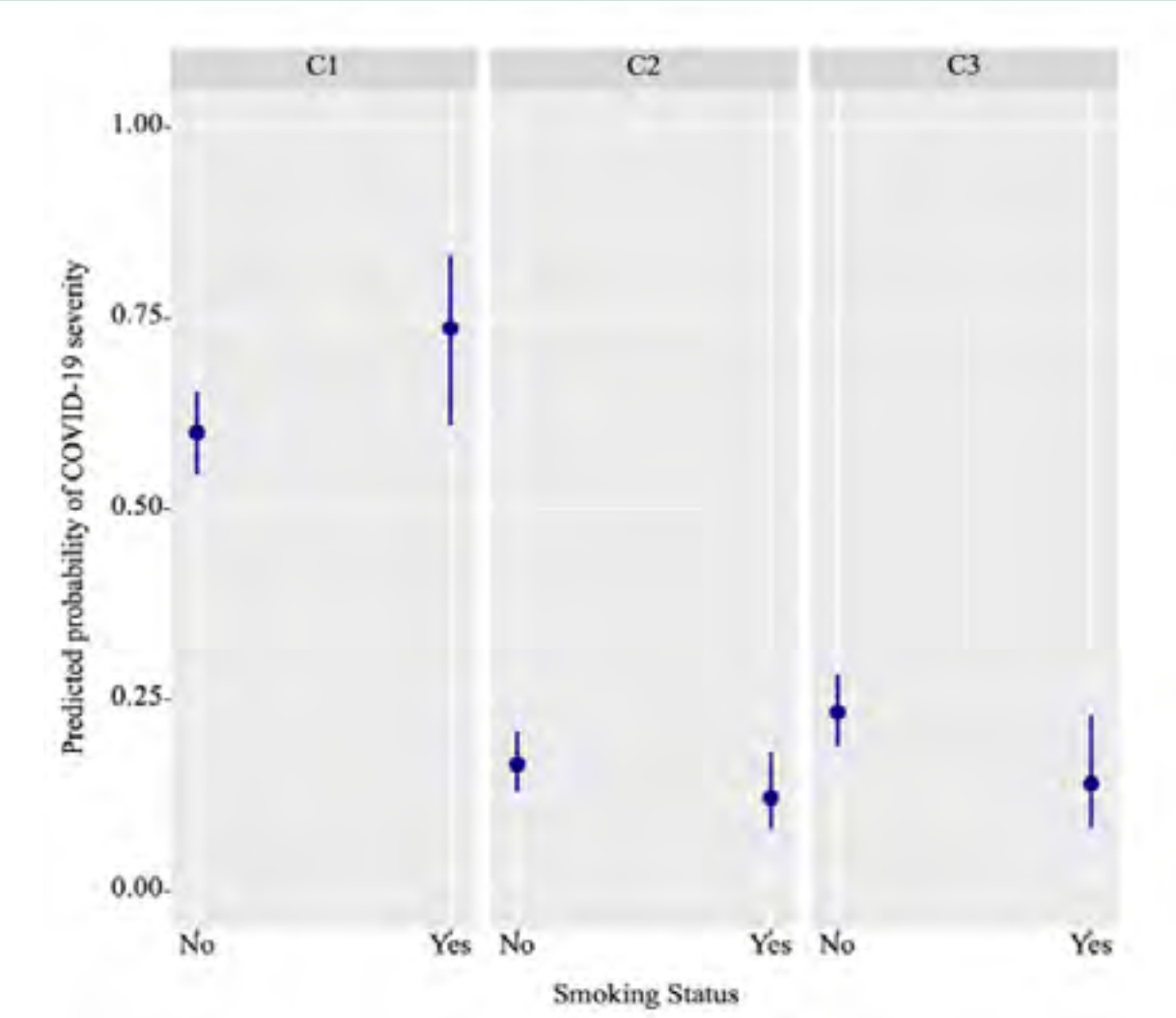

Figure S3. Model-predicted probabilities of COVID-19 severity categories according to smoking status. Points are predicted probabilities and vertical bars the 95% confidence intervals. C1, mild; C2, moderate; C3, severe/critical.

© 2026 by the authors. This article is an open access article distributed under the terms and conditions of the Creative Commons Attribution (CC BY 4.0) license.

Table S4. Ordinal logistic regression analysis of the association between SpO2 at onset and COVID-19 severity

Parameter	OR	95% CI	*P*
SpO_2 at onset (%)	0.45	0.37–0.54	**< 0.001**
Observations (*n*)	206		
Nagelkerke R^2	0.697		

Estimates are odds ratios from a univariable ordinal logistic regression model with COVID-19 severity as the ordered outcome (C1, mild; C2, moderate; C3, severe/critical). Bold *P* values indicate statistical significance ($P < 0.05$). CI, confidence interval; *n*, number of patients contributing to the model; OR, odds ratio; SpO2, peripheral oxygen saturation.

Table S5. Ordinal logistic regression analysis of the association between eosinophil count at onset and COVID-19 severity

Parameter	OR	95% CI	*P*
1|2	0.64	0.39–1.07	0.091
2|3	1.64	0.98–2.74	0.058
Eosinophils (×10^9/L)	0.50	0.30–0.86	**0.012**
Observations (*n*)	108		
Nagelkerke R^2	0.092		

Estimates are odds ratios from a univariable ordinal logistic regression model with COVID-19 severity as the ordered outcome (C1, mild; C2, moderate; C3, severe/critical). Rows 1|2 and 2|3 are the threshold (intercept) parameters for the severity cut-points. Bold *P* values indicate statistical significance ($P < 0.05$). CI, confidence interval; *n*, number of patients contributing to the model; OR, odds ratio.

Table S6. Ordinal logistic regression analysis of the association between SIRI and COVID-19 severity

Parameter	OR	95% CI	*P*
1|2	1.29	0.80–2.06	0.290
2|3	2.92	1.74–4.91	**< 0.001**
SIRI	1.00	1.00–1.00	0.701
Observations (*n*)	108		

Estimates are odds ratios from a univariable ordinal logistic regression model with COVID-19 severity as the ordered outcome (C1, mild; C2, moderate; C3, severe/critical). Rows 1|2 and 2|3 are the threshold (intercept) parameters for the severity cut-points. Bold *P* values indicate statistical significance ($P < 0.05$). CI, confidence interval; *n*, number of patients contributing to the model; OR, odds ratio; SIRI, systemic inflammation response index.

Table S7. Ordinal logistic regression analysis of the association between AISI and COVID-19 severity

Parameter	OR	95% CI	*P*
1|2	1.38	0.81–2.36	0.229
2|3	3.17	1.76–5.70	**< 0.001**
AISI	1.00	1.00–1.00	0.533
Observations (*n*)	108		

Estimates are odds ratios from a univariable ordinal logistic regression model with COVID-19 severity as the ordered outcome (C1, mild; C2, moderate; C3, severe/critical). Rows 1|2 and 2|3 are the threshold (intercept) parameters for the severity cut-points. Bold *P* values indicate statistical significance ($P < 0.05$). CI, confidence interval; *n*, number of patients contributing to the model; OR, odds ratio; AISI, aggregate index of systemic inflammation.

Table S8. Ordinal logistic regression analysis of the association between HCT and COVID-19 severity

Parameter	OR	95% CI	*P*
1|2	0.04	0.00–0.54	**0.016**
2|3	0.10	0.01–1.25	0.073
HCT (%)	0.91	0.85–0.98	**0.010**
Observations (*n*)	108		
Nagelkerke R^2	0.076		

Estimates are odds ratios from a univariable ordinal logistic regression model with COVID-19 severity as the ordered outcome (C1, mild; C2, moderate; C3, severe/critical). Rows 1|2 and 2|3 are the threshold (intercept) parameters for the severity cut-points. Bold *P* values indicate statistical significance ($P < 0.05$). CI, confidence interval; *n*, number of patients contributing to the model; OR, odds ratio; HCT, hematocrit.

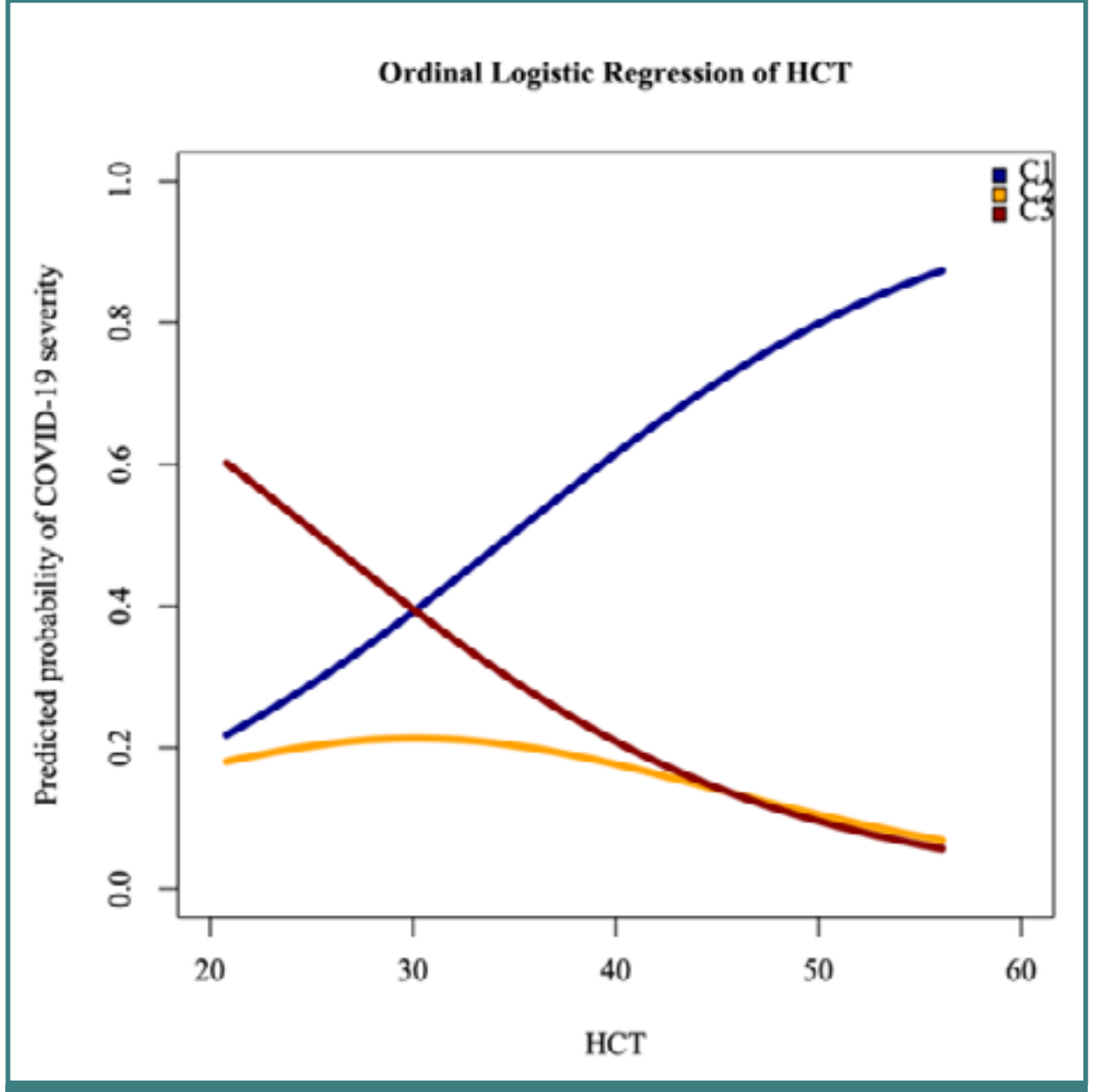

Figure S4. Model-predicted probabilities of COVID-19 severity categories across HCT values. C1, mild; C2, moderate; C3, severe/critical; HCT, hematocrit.

© 2026 by the authors. This article is an open access article distributed under the terms and conditions of the Creative Commons Attribution (CC BY 4.0) license.

Table S9. Ordinal logistic regression analysis of the association between AST (GOT) and COVID-19 severity

Parameter	OR	95% CI	*P*
1|2	2.22	1.23–4.02	**0.009**
2|3	5.36	2.75–10.44	**< 0.001**
AST (GOT) (U/L)	1.02	1.00–1.03	**0.010**
Observations (*n*)	108		
Nagelkerke R^2	0.091		

Estimates are odds ratios from a univariable ordinal logistic regression model with COVID-19 severity as the ordered outcome (C1, mild; C2, moderate; C3, severe/critical). Rows 1|2 and 2|3 are the threshold (intercept) parameters for the severity cut-points. Bold *P* values indicate statistical significance ($P < 0.05$). CI, confidence interval; *n*, number of patients contributing to the model; OR, odds ratio; AST, aspartate aminotransferase; GOT, glutamic-oxaloacetic transaminase.

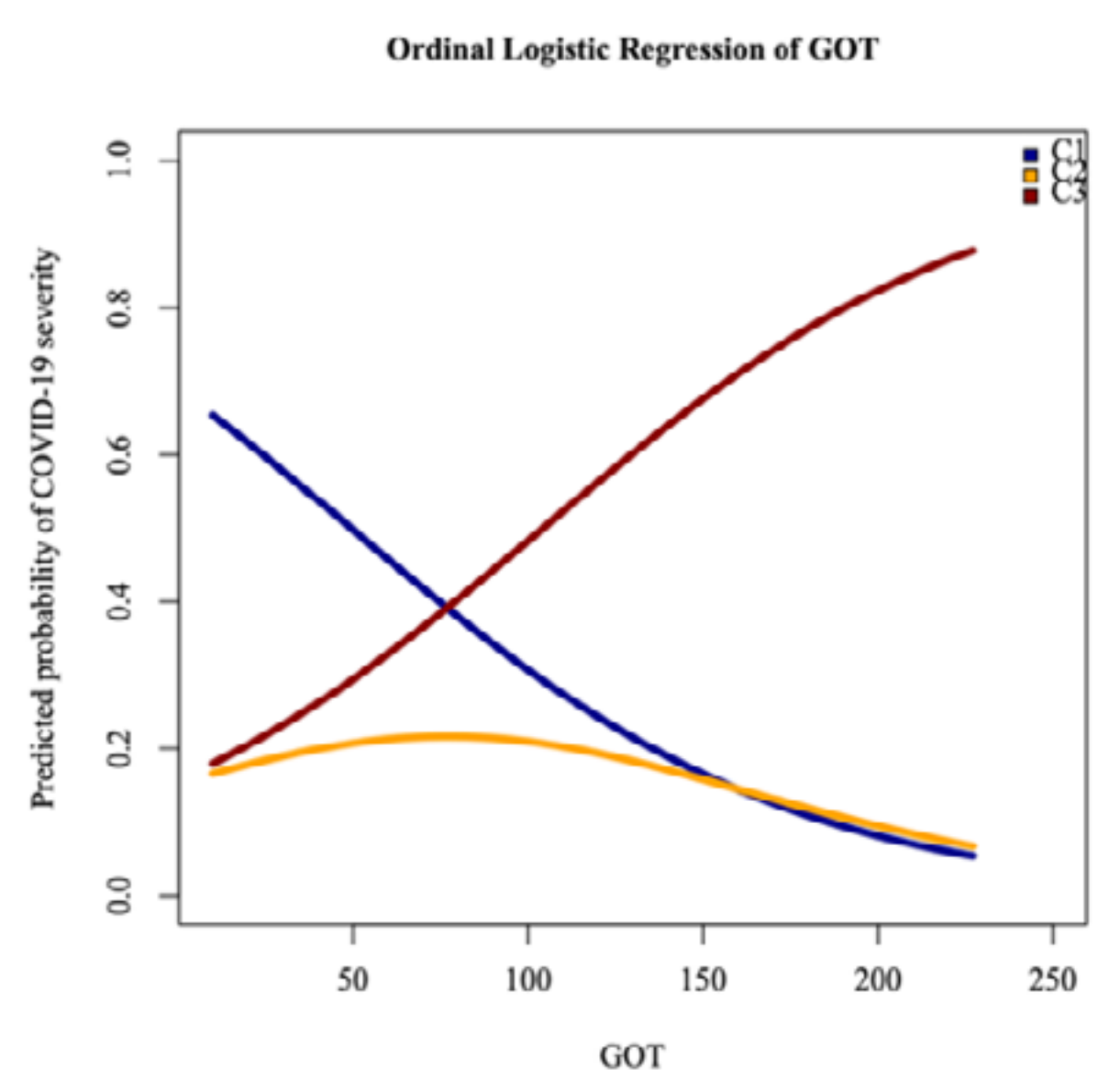

Figure S5. Model-predicted probabilities of COVID-19 severity categories across AST (GOT) values. C1, mild; C2, moderate; C3, severe/critical; AST, aspartate aminotransferase; GOT, glutamic-oxaloacetic transaminase.

Table S10. Ordinal logistic regression analysis of the association between procalcitonin and COVID-19 severity

Parameter	OR	95% CI	*P*
1|2	0.46	0.20–1.05	0.065
2|3	1.93	0.88–4.24	0.097
Procalcitonin (ng/mL)	3.12	0.58–16.88	0.180
Observations (*n*)	44		

Estimates are odds ratios from a univariable ordinal logistic regression model with COVID-19 severity as the ordered outcome (C1, mild; C2, moderate; C3, severe/critical). Rows 1|2 and 2|3 are the threshold (intercept) parameters for the severity cut-points. Bold *P* values indicate statistical significance ($P < 0.05$). CI, confidence interval; *n*, number of patients contributing to the model; OR, odds ratio.

Table S11. Ordinal logistic regression analysis of the association between D-dimers and COVID-19 severity

Parameter	OR	95% CI	*P*
1|2	0.60	0.31–1.16	0.128
2|3	1.98	1.02–3.84	**0.045**
D-dimers (ng/mL)	1.00	1.00–1.00	0.909
Observations (*n*)	73		

Estimates are odds ratios from a univariable ordinal logistic regression model with COVID-19 severity as the ordered outcome (C1, mild; C2, moderate; C3, severe/critical). Rows 1|2 and 2|3 are the threshold (intercept) parameters for the severity cut-points. Bold *P* values indicate statistical significance ($P < 0.05$). CI, confidence interval; *n*, number of patients contributing to the model; OR, odds ratio.

Table S12. Ordinal logistic regression analysis of the association between hemoglobin and COVID-19 severity

Parameter	OR	95% CI	*P*
1|2	0.18	0.02–1.61	0.124
2|3	0.45	0.05–3.96	0.471
Hemoglobin (g/dL)	0.87	0.73–1.03	0.112
Observations (*n*)	110		

Estimates are odds ratios from a univariable ordinal logistic regression model with COVID-19 severity as the ordered outcome (C1, mild; C2, moderate; C3, severe/critical). Rows 1|2 and 2|3 are the threshold (intercept) parameters for the severity cut-points. Bold *P* values indicate statistical significance ($P < 0.05$). CI, confidence interval; *n*, number of patients contributing to the model; OR, odds ratio.

Table S13. Ordinal logistic regression analysis of the association between platelet count and COVID-19 severity

Parameter	OR	95% CI	*P*
1|2	0.70	0.23–2.15	0.530
2|3	1.60	0.52–4.91	0.412
Platelet count ($\times 10^9$/L)	1.00	0.99–1.00	0.316
Observations (*n*)	108		

Estimates are odds ratios from a univariable ordinal logistic regression model with COVID-19 severity as the ordered outcome (C1, mild; C2, moderate; C3, severe/critical). Rows 1|2 and 2|3 are the threshold (intercept) parameters for the severity cut-points. Bold *P* values indicate statistical significance ($P < 0.05$). CI, confidence interval; *n*, number of patients contributing to the model; OR, odds ratio.

© 2026 by the authors. This article is an open access article distributed under the terms and conditions of the Creative Commons Attribution (CC BY 4.0) license.

Table S14. Ordinal logistic regression analysis of the association between total leukocyte count and COVID-19 severity

Parameter	OR	95% CI	*P*
1|2	1.16	0.53–2.51	0.708
2|3	2.85	1.28–6.34	**0.011**
Total leukocyte count (×10^9/L)	1.01	0.93–1.11	0.746
Observations (*n*)	110		

Estimates are odds ratios from a univariable ordinal logistic regression model with COVID-19 severity as the ordered outcome (C1, mild; C2, moderate; C3, severe/critical). Rows 1|2 and 2|3 are the threshold (intercept) parameters for the severity cut-points. Bold *P* values indicate statistical significance ($P < 0.05$). CI, confidence interval; *n*, number of patients contributing to the model; OR, odds ratio.

Table S15. Ordinal logistic regression analysis of the association between ALT (GPT) and COVID-19 severity

Parameter	OR	95% CI	*P*
1|2	1.49	0.78–2.83	0.221
2|3	3.38	1.71–6.68	**0.001**
ALT (GPT) (U/L)	1.01	0.99–1.02	0.411
Observations (*n*)	108		

Estimates are odds ratios from a univariable ordinal logistic regression model with COVID-19 severity as the ordered outcome (C1, mild; C2, moderate; C3, severe/critical). Rows 1|2 and 2|3 are the threshold (intercept) parameters for the severity cut-points. Bold *P* values indicate statistical significance ($P < 0.05$). CI, confidence interval; *n*, number of patients contributing to the model; OR, odds ratio; ALT, alanine aminotransferase; GPT, glutamic-pyruvic transaminase.

Table S16. Ordinal logistic regression analysis of the association between CK and COVID-19 severity

Parameter	OR	95% CI	*P*
1|2	0.61	0.33–1.12	0.108
2|3	1.92	1.03–3.55	**0.040**
CK (U/L)	1.00	1.00–1.00	0.755
Observations (*n*)	54		

Estimates are odds ratios from a univariable ordinal logistic regression model with COVID-19 severity as the ordered outcome (C1, mild; C2, moderate; C3, severe/critical). Rows 1|2 and 2|3 are the threshold (intercept) parameters for the severity cut-points. Bold *P* values indicate statistical significance ($P < 0.05$). CI, confidence interval; *n*, number of patients contributing to the model; OR, odds ratio; CK, creatine kinase.

Table S17. Ordinal logistic regression analysis of the association between serum creatinine and COVID-19 severity

Parameter	OR	95% CI	*P*
1|2	1.31	0.58–2.98	0.514
2|3	2.97	1.27–6.93	**0.012**
Serum creatinine (mg/dL)	1.09	0.51–2.35	0.816
Observations (*n*)	108		

Estimates are odds ratios from a univariable ordinal logistic regression model with COVID-19 severity as the ordered outcome (C1, mild; C2, moderate; C3, severe/critical). Rows 1|2 and 2|3 are the threshold (intercept) parameters for the severity cut-points. Bold *P* values indicate statistical significance ($P < 0.05$). CI, confidence interval; *n*, number of patients contributing to the model; OR, odds ratio.

Table S18. Ordinal logistic regression analysis of the association between arterial hypertension and COVID-19 severity

Parameter	OR	95% CI	*P*
1|2	0.99	0.67–1.46	0.957
2|3	2.61	1.71–3.97	**< 0.001**
Absence of arterial hypertension (vs presence)	0.32	0.18–0.56	**< 0.001**
Observations (*n*)	211		

Estimates are odds ratios from a univariable ordinal logistic regression model with COVID-19 severity as the ordered outcome (C1, mild; C2, moderate; C3, severe/critical). Rows 1|2 and 2|3 are the threshold (intercept) parameters for the severity cut-points. Bold *P* values indicate statistical significance ($P < 0.05$). CI, confidence interval; *n*, number of patients contributing to the model; OR, odds ratio.

Table S19. Ordinal logistic regression analysis of the association between diabetes mellitus and COVID-19 severity

Parameter	OR	95% CI	*P*
1|2	0.52	0.24–1.11	0.090
2|3	1.36	0.64–2.87	0.425
Absence of diabetes mellitus (vs presence)	0.25	0.11–0.55	**0.001**
Observations (*n*)	211		

Estimates are odds ratios from a univariable ordinal logistic regression model with COVID-19 severity as the ordered outcome (C1, mild; C2, moderate; C3, severe/critical). Rows 1|2 and 2|3 are the threshold (intercept) parameters for the severity cut-points. Bold *P* values indicate statistical significance ($P < 0.05$). CI, confidence interval; *n*, number of patients contributing to the model; OR, odds ratio.

© 2026 by the authors. This article is an open access article distributed under the terms and conditions of the Creative Commons Attribution (CC BY 4.0) license.

Table S20. Ordinal logistic regression analysis of the association between obesity and COVID-19 severity

Parameter	OR	95% CI	*P*
1|2	1.33	0.68–2.60	0.410
2|3	3.30	1.65–6.62	**0.001**
Absence of obesity (vs presence)	0.71	0.34–1.47	0.352
Observations (*n*)	211		

Estimates are odds ratios from a univariable ordinal logistic regression model with COVID-19 severity as the ordered outcome (C1, mild; C2, moderate; C3, severe/critical). Rows 1|2 and 2|3 are the threshold (intercept) parameters for the severity cut-points. Bold *P* values indicate statistical significance ($P < 0.05$). CI, confidence interval; *n*, number of patients contributing to the model; OR, odds ratio.

Table S21. Ordinal logistic regression analysis of the association between thyroid dysfunction and COVID-19 severity

Parameter	OR	95% CI	*P*
1|2	0.96	0.55–1.68	0.890
2|3	2.44	1.37–4.35	**0.002**
Absence of thyroid dysfunction (vs presence)	0.45	0.24–0.85	0.015
Observations (*n*)	211		

Estimates are odds ratios from a univariable ordinal logistic regression model with COVID-19 severity as the ordered outcome (C1, mild; C2, moderate; C3, severe/critical). Rows 1|2 and 2|3 are the threshold (intercept) parameters for the severity cut-points. Bold *P* values indicate statistical significance ($P < 0.05$). CI, confidence interval; *n*, number of patients contributing to the model; OR, odds ratio.

Table S22. Ordinal logistic regression analysis of the association between pulmonary disease and COVID-19 severity

Parameter	OR	95% CI	*P*
1|2	1.39	0.86–2.25	0.185
2|3	3.46	2.06–5.81	**< 0.001**
Absence of pulmonary disease (vs presence)	0.70	0.39–1.25	0.225
Observations (*n*)	211		

Estimates are odds ratios from a univariable ordinal logistic regression model with COVID-19 severity as the ordered outcome (C1, mild; C2, moderate; C3, severe/critical). Rows 1|2 and 2|3 are the threshold (intercept) parameters for the severity cut-points. Bold *P* values indicate statistical significance ($P < 0.05$). CI, confidence interval; *n*, number of patients contributing to the model; OR, odds ratio.

Table S23. Ordinal logistic regression analysis of the association between malignancy and COVID-19 severity

Parameter	OR	95% CI	*P*
1|2	0.71	0.29–1.73	0.453
2|3	1.80	0.74–4.39	0.197
Absence of malignancy (vs presence)	0.37	0.15–0.93	**0.035**
Observations (*n*)	211		

Estimates are odds ratios from a univariable ordinal logistic regression model with COVID-19 severity as the ordered outcome (C1, mild; C2, moderate; C3, severe/critical). Rows 1|2 and 2|3 are the threshold (intercept) parameters for the severity cut-points. Bold *P* values indicate statistical significance ($P < 0.05$). CI, confidence interval; *n*, number of patients contributing to the model; OR, odds ratio.

Table S24. Ordinal logistic regression analysis of the association between osteoporosis and COVID-19 severity

Parameter	OR	95% CI	*P*
1|2	1.39	0.81–2.40	0.235
2|3	3.47	1.95–6.17	**< 0.001**
Absence of osteoporosis (vs presence)	0.72	0.39–1.36	0.314
Observations (*n*)	211		

Estimates are odds ratios from a univariable ordinal logistic regression model with COVID-19 severity as the ordered outcome (C1, mild; C2, moderate; C3, severe/critical). Rows 1|2 and 2|3 are the threshold (intercept) parameters for the severity cut-points. Bold *P* values indicate statistical significance ($P < 0.05$). CI, confidence interval; *n*, number of patients contributing to the model; OR, odds ratio.

Table S25. Ordinal logistic regression analysis of the association between neurologic disease and COVID-19 severity

Parameter	OR	95% CI	*P*
1|2	0.87	0.32–2.42	0.795
2|3	2.19	0.78–6.10	0.135
Absence of neurologic disease (vs presence)	0.47	0.16–1.34	0.158
Observations (*n*)	211		

Estimates are odds ratios from a univariable ordinal logistic regression model with COVID-19 severity as the ordered outcome (C1, mild; C2, moderate; C3, severe/critical). Rows 1|2 and 2|3 are the threshold (intercept) parameters for the severity cut-points. Bold *P* values indicate statistical significance ($P < 0.05$). CI, confidence interval; *n*, number of patients contributing to the model; OR, odds ratio.

© 2026 by the authors. This article is an open access article distributed under the terms and conditions of the Creative Commons Attribution (CC BY 4.0) license.
