# Supplementary material for: COVID-19 severity and early inflammatory biomarkers in patients with immune-mediated inflammatory rheumatic diseases: a multicenter cohort study
Source: J Med Life. 2026 Aug;19(8):657–71. doi: 10.25122/jml-2026-0098 (PMC13674051; doi:10.25122/jml-2026-0098)
Supplement: Supplementary file 4 [file JMedLife-19-657-s004.pdf]

Supplementary Material 4. Missingness patterns for laboratory variables in the overall cohort and according to COVID-19 severity category

Biomarker	Overall cohort (*n* = 211)	C1 (*n* = 135)	C2 (*n* = 37)	C3 (*n* = 39)
ESR	130 (61.6%)	96 (71.1%)	16 (43.2%)	18 (46.2%)
CRP	103 (48.8%)	78 (57.8%)	13 (35.1%)	12 (30.8%)
Fibrinogen	152 (72.0%)	117 (86.7%)	21 (56.8%)	14 (35.9%)
Lymphocytes	101 (47.9%)	79 (58.5%)	14 (37.8%)	8 (20.5%)
Monocytes	103 (48.8%)	80 (59.3%)	15 (40.5%)	8 (20.5%)
Eosinophils	103 (48.8%)	80 (59.3%)	15 (40.5%)	8 (20.5%)
NLR	103 (48.8%)	76 (56.3%)	17 (45.9%)	10 (25.6%)
dNLR	103 (48.8%)	76 (56.3%)	17 (45.9%)	10 (25.6%)
SIRI	103 (48.8%)	76 (56.3%)	17 (45.9%)	10 (25.6%)
AISI	103 (48.8%)	76 (56.3%)	17 (45.9%)	10 (25.6%)
HCT	103 (48.8%)	76 (56.3%)	17 (45.9%)	10 (25.6%)
AST	103 (48.8%)	76 (56.3%)	17 (45.9%)	10 (25.6%)
Procalcitonin	167 (79.1%)	124 (91.9%)	23 (62.2%)	20 (51.3%)
D-dimers	138 (65.4%)	108 (80.0%)	16 (43.2%)	14 (35.9%)
Hemoglobin	101 (47.9%)	79 (58.5%)	14 (37.8%)	8 (20.5%)
Platelet count	103 (48.8%)	76 (56.3%)	17 (45.9%)	10 (25.6%)
Total leukocyte count	101 (47.9%)	79 (58.5%)	14 (37.8%)	8 (20.5%)
ALT	103 (48.8%)	76 (56.3%)	17 (45.9%)	10 (25.6%)
CK	157 (74.4%)	115 (85.2%)	22 (59.5%)	20 (51.3%)
Serum creatinine	103 (48.8%)	76 (56.3%)	17 (45.9%)	10 (25.6%)

Severity categories: C1, mild; C2, moderate; C3, severe/critical. AISI, aggregate index of systemic inflammation; ALT, alanine aminotransferase; AST, aspartate aminotransferase; CK, creatine kinase; CRP, C-reactive protein; dNLR, derived neutrophil-to-lymphocyte ratio; ESR, erythrocyte sedimentation rate; HCT, hematocrit; *n*, number of patients; NLR, neutrophil-to-lymphocyte ratio; SIRI, systemic inflammation response index.

© 2026 by the authors. This article is an open access article distributed under the terms and conditions of the Creative Commons Attribution (CC BY 4.0) license.
