# Supplementary material for: COVID-19 severity and early inflammatory biomarkers in patients with immune-mediated inflammatory rheumatic diseases: a multicenter cohort study
Source: J Med Life. 2026 Aug;19(8):657–71. doi: 10.25122/jml-2026-0098 (PMC13674051; doi:10.25122/jml-2026-0098)
Supplement: Supplementary file 5 [file JMedLife-19-657-s005.pdf]

Supplementary Material 5. Bonferroni-adjusted *P* values for the biomarkers evaluated in the univariable ordinal logistic regression analyses

**Parameter**	**Raw *P***	**Calculation**	**Adjusted *P***
ESR	0.001	0.001 × 20 = 0.020	**0.020**
CRP	0.001	0.001 × 20 = 0.020	**0.020**
Fibrinogen	0.002	0.002 × 20 = 0.040	**0.040**
Lymphocytes	0.003	0.003 × 20 = 0.060	0.060
Monocytes	0.001	0.001 × 20 = 0.020	**0.020**
Eosinophils	0.012	0.012 × 20 = 0.240	0.240
NLR	0.006	0.006 × 20 = 0.120	0.120
dNLR	0.002	0.002 × 20 = 0.040	**0.040**
SIRI	0.701	0.701 × 20 = 14.02	1.000
AISI	0.533	0.533 × 20 = 10.66	1.000
HCT	0.010	0.010 × 20 = 0.200	0.200
AST	0.010	0.010 × 20 = 0.200	0.200
Procalcitonin	0.180	0.180 × 20 = 3.60	1.000
D-dimers	0.909	0.909 × 20 = 18.18	1.000
Hemoglobin	0.112	0.112 × 20 = 2.24	1.000
Platelet count	0.316	0.316 × 20 = 6.32	1.000
Total leukocyte count	0.746	0.746 × 20 = 14.92	1.000
ALT	0.411	0.411 × 20 = 8.22	1.000
CK	0.755	0.755 × 20 = 15.10	1.000
Serum creatinine	0.816	0.816 × 20 = 16.32	1.000

Bonferroni-adjusted *P* values were calculated by multiplying each raw *P* value by 20, the number of laboratory parameters evaluated, with products exceeding 1.00 capped at 1.00. Bold adjusted *P* values indicate statistical significance (adjusted $P < 0.05$). AISI, aggregate index of systemic inflammation; ALT, alanine aminotransferase; AST, aspartate aminotransferase; CK, creatine kinase; CRP, C-reactive protein; dNLR, derived neutrophil-to-lymphocyte ratio; ESR, erythrocyte sedimentation rate; HCT, hematocrit; NLR, neutrophil-to-lymphocyte ratio; SIRI, systemic inflammation response index.

© 2026 by the authors. This article is an open access article distributed under the terms and conditions of the Creative Commons Attribution (CC BY 4.0) license.
